# Supplementary material for: New Pyridinium Compound from Marine Sediment-Derived Bacterium Bacillus licheniformis S-1
Source: Molecules. 2024 Dec 24;30(1):7. doi: 10.3390/molecules30010007 (PMC11722202; doi:10.3390/molecules30010007)
Supplement: Supplementary file 1 [file molecules-30-00007-s001.zip › molecules-3338296-supplementary.pdf]

Catalogue

Figure S1. HR-ESI-MS of compound **1**. 3
Figure S2.^{1}H NMR spectrum (600 MHz, MeOD) of compound **1**. 3
Figure S3.^{13}C NMR and DEPT spectra (151 MHz, MeOD) of compound **1**. 3
Figure S4. HSQC spectrum (600 MHz, MeOD) of compound **1**. 4
Figure S5.^{1}H-^{1}H COSY spectrum (600 MHz, MeOD) of compound **1**. 4
Figure S6. HMBC spectrum (400 MHz, CD_3OD) of compound **1**. 5
Figure S7. HR-ESI-MS of compound **2**........ 5
Figure S8.^{1}H NMR spectrum (600 MHz, $CDCl_3$) of compound **2**........ 6
Figure S9.^{13}C NMR spectrum (151 MHz, $CDCl_3$) of compound **2**........ 6
Figure S10. HR-ESI-MS of compound **3** 7
Figure S11.^{1}H NMR spectrum (600 MHz, MeOD) of compound **3**. 7
Figure S12.^{13}C NMR and DEPT spectra (151 MHz, MeOD) of compound **3**. 8
Figure S13. HR-ESI-MS of compound **4** 8
Figure S14.^{1}H NMR spectrum (600 MHz, $CDCl_3$) of compound **4**. 9
Figure S15.^{13}C NMR spectrum (151 MHz, $CDCl_3$) of compound **4**. 9
Figure S16. HR-ESI-MS of compound **5** 10
Figure S17.^{1}H NMR spectrum (600 MHz, MeOD) of compound **5**. 10
Figure S18.^{13}C NMR spectrum (151 MHz, MeOD) of compound **5**. 11
Figure S19. HR-ESI-MS of compound **6** 11
Figure S20.^{1}H NMR spectrum (600 MHz, MeOD) of compound **6**. 12
Figure S21.^{13}C NMR and DEPT spectra (151 MHz, MeOD) of compound **6**. 12
Figure S22. HR-ESI-MS of compound **7** 13
Figure S23.^{1}H NMR spectrum (600 MHz, MeOD) of compound **7**. 13
Figure S24.^{13}C NMR and DEPT spectra (151 MHz, MeOD) of compound **7**. 14
Figure S25. HR-ESI-MS of compound **8** 14
Figure S26.^{1}H NMR spectrum (600 MHz, MeOD) of compound **8**. 15
Figure S27.^{13}C NMR and DEPT spectra (151 MHz, MeOD) of compound **8**. 15
Figure S28. HR-ESI-MS of compound **9** 16
Figure S29.^{1}H NMR spectrum (600 MHz, MeOD) of compound **9** 16
Figure S30.^{13}C NMR and DEPT spectra (151 MHz, MeOD) of compound **9**. 17
Figure S31. HR-ESI-MS of compound **10** 17
Figure S32.^{1}H NMR spectrum (600 MHz, MeOD) of compound **10**........ 18
Figure S33.^{13}C NMR and DEPT spectra (151 MHz, MeOD) of compound **10**. 18
Figure S34.^{1}H NMR spectrum (600 MHz, MeOD) of compound **11**. 19
Figure S35.^{13}C NMR and DEPT spectra (151 MHz, MeOD) of compound **11**. 19
Figure S36. HR-ESI-MS of compound **12** 20
Figure S37.^{1}H NMR spectrum (600 MHz, MeOD) of compound **12**........ 20
Figure S38.^{13}C NMR and DEPT spectra (151 MHz, MeOD) of compound **12**. 21
Figure S39. HR-ESI-MS of compound **13** 21
Figure S40.^{1}H NMR spectrum (600 MHz, MeOD) of compound **13**........ 22
Figure S41.^{13}C NMR and DEPT spectra (151 MHz, MeOD) of compound **13**. 22
Figure S42. HR-ESI-MS of compound **14** 23

Figure S43.^{1}H NMR spectrum (600 MHz, MeOD) of compound **14**. 23
Figure S44.^{13}C NMR and DEPT spectra (151 MHz, MeOD) of compound **14**. 24
Figure S45. HR-ESI-MS of compound **15** 24
Figure S46.^{1}H NMR spectrum (600 MHz, MeOD) of compound **15**. 25
Figure S47.^{13}C NMR and DEPT spectra (151 MHz, MeOD) of compound **15**. 25
Figure S48. HR-ESI-MS of compound **16** 26
Figure S49.^{1}H NMR spectrum (600 MHz, MeOD) of compound **16**. 26
Figure S50.^{13}C NMR and DEPT spectra (151 MHz, MeOD) of compound **16**. 27
Figure S51. The graphical representation of the separation process. 27
Table S1. Specific OR of compounds **1–16**. 29
Table S2. Antibacterial activity of compound **1-16** (50μg/mL). 29
Table S3. Antifungal activity of compound **1-16** (50μg/mL). 31
Table S4. Acronym list. 31

Figure S1. HR-ESI-MS of compound **1**.

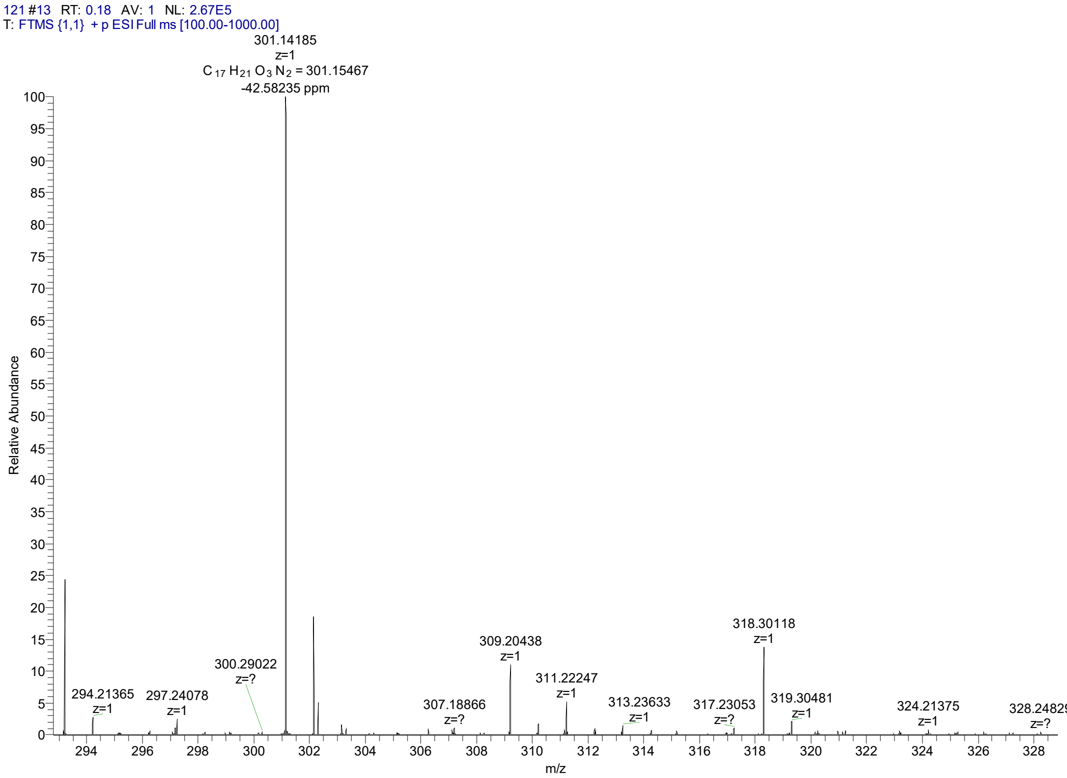

Figure S2.^{1}H NMR spectrum (600 MHz, MeOD) of compound **1**.

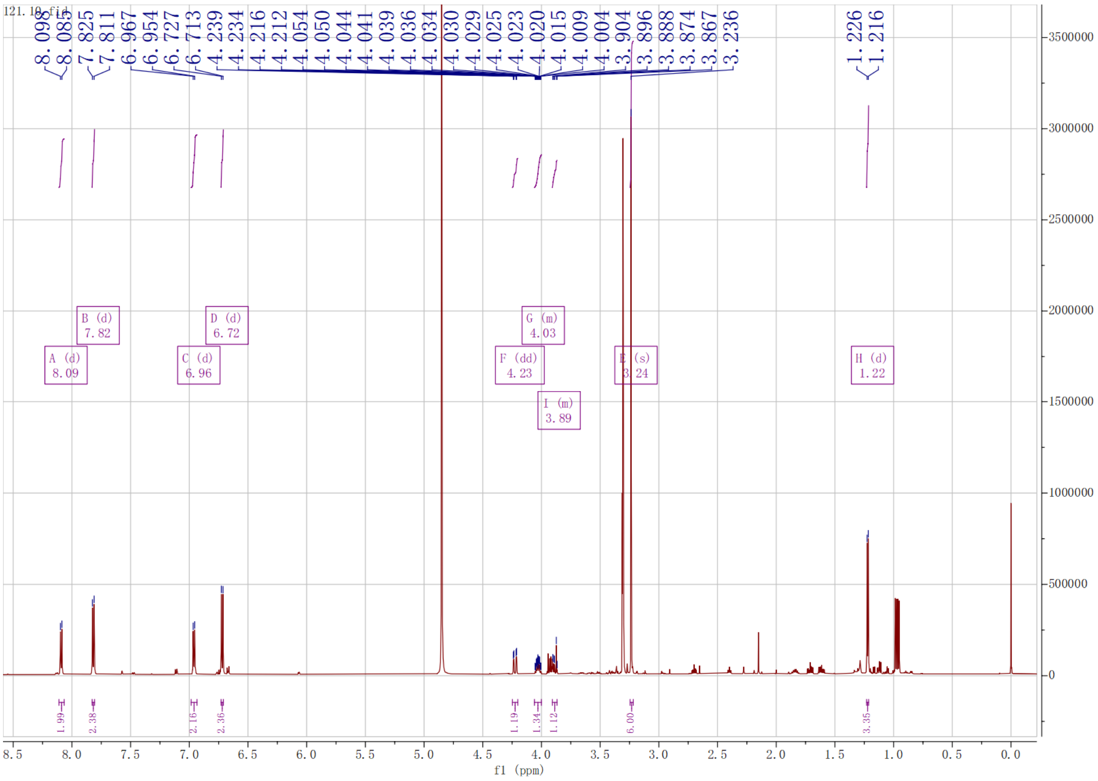

Figure S3.^{13}C NMR and DEPT spectra (151 MHz, MeOD) of compound **1**.

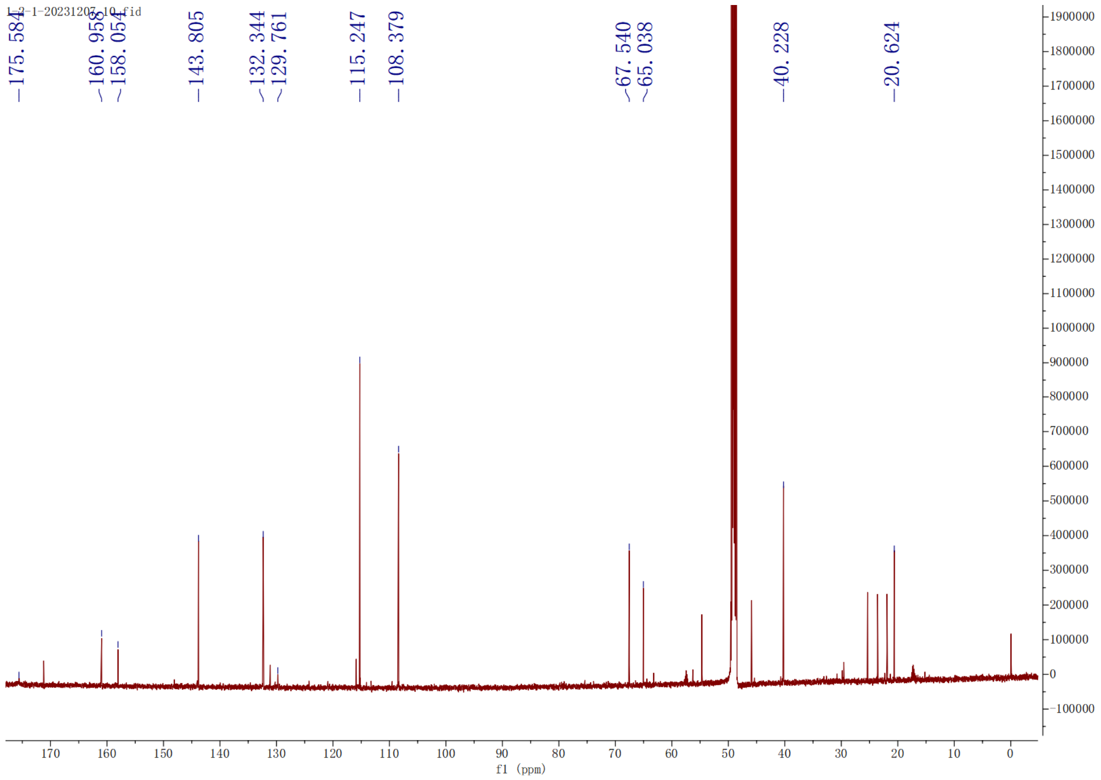

Figure S4. HSQC spectrum (600 MHz, MeOD) of compound **1**.

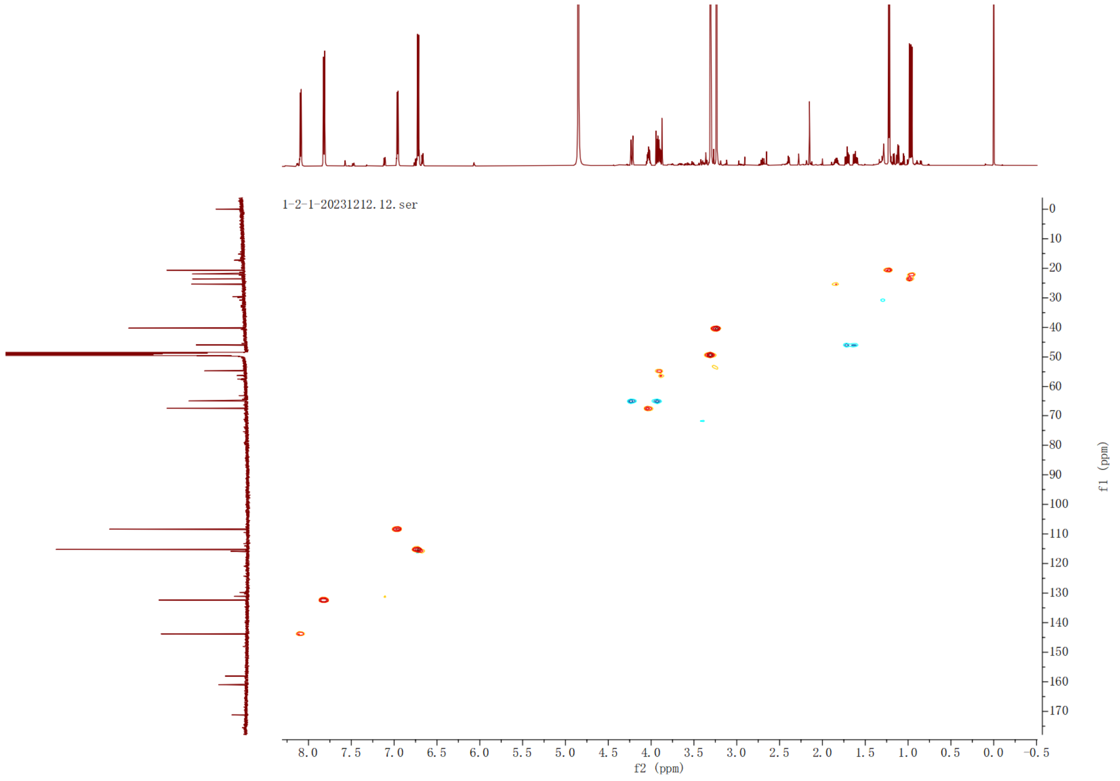

Figure S5. ^{1}H-^{1}H COSY spectrum (600 MHz, MeOD) of compound **1**.

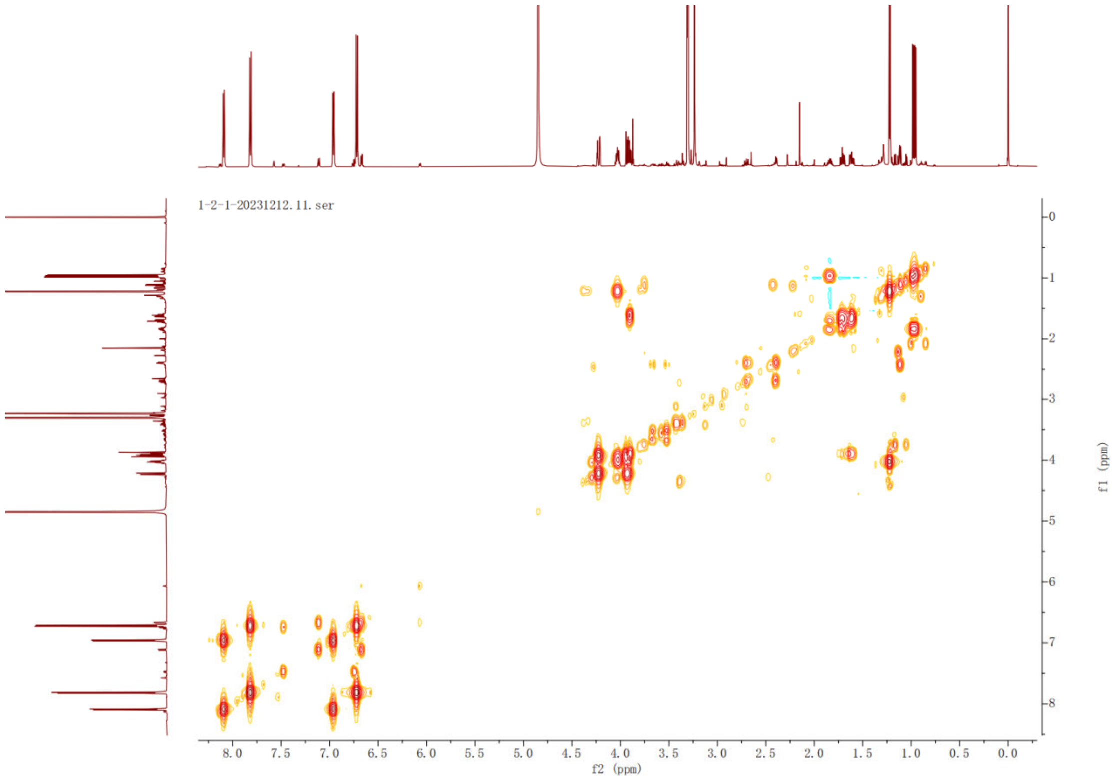

Figure S6. HMBC spectrum (400 MHz, CD_3OD) of compound **1**.

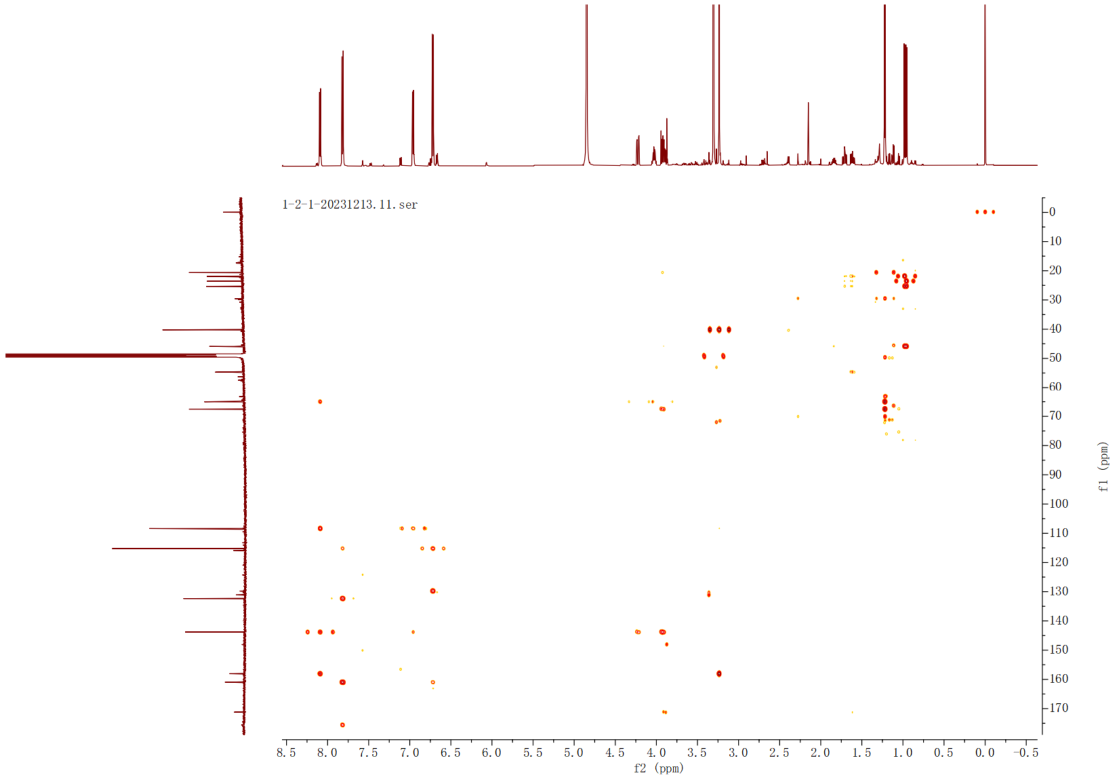

Figure S7. HR-ESI-MS of compound **2**

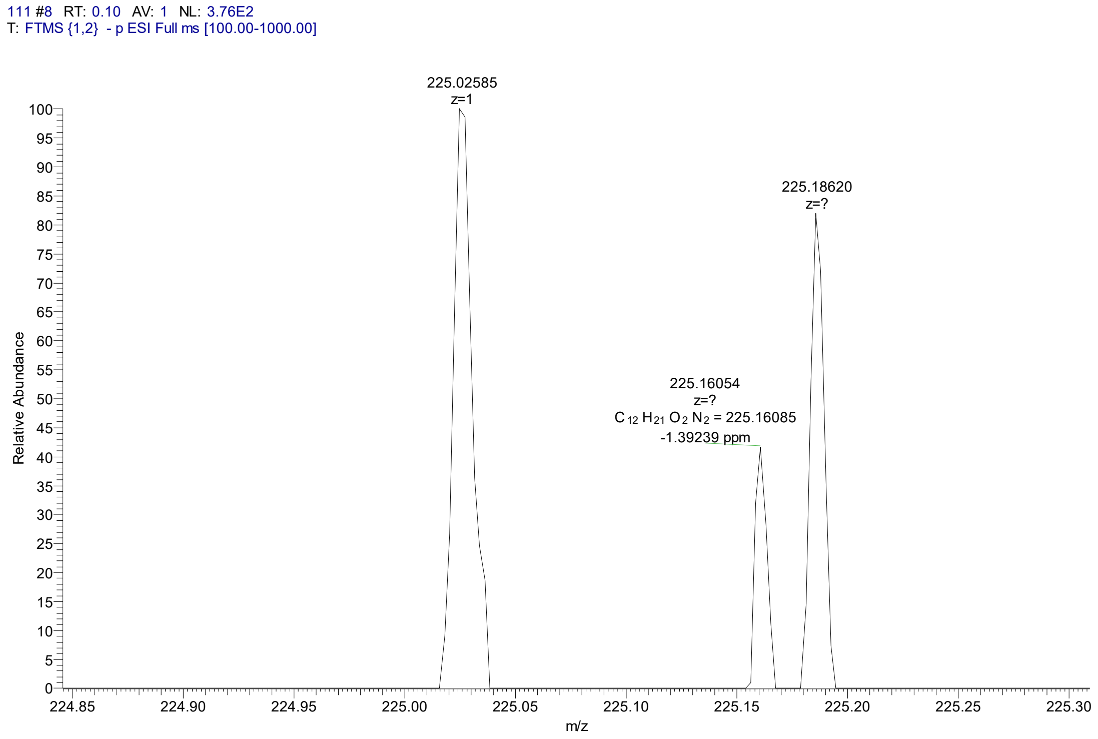

Figure S8. ^{1}H NMR spectrum (600 MHz, $CDCl_3$) of compound **2**.

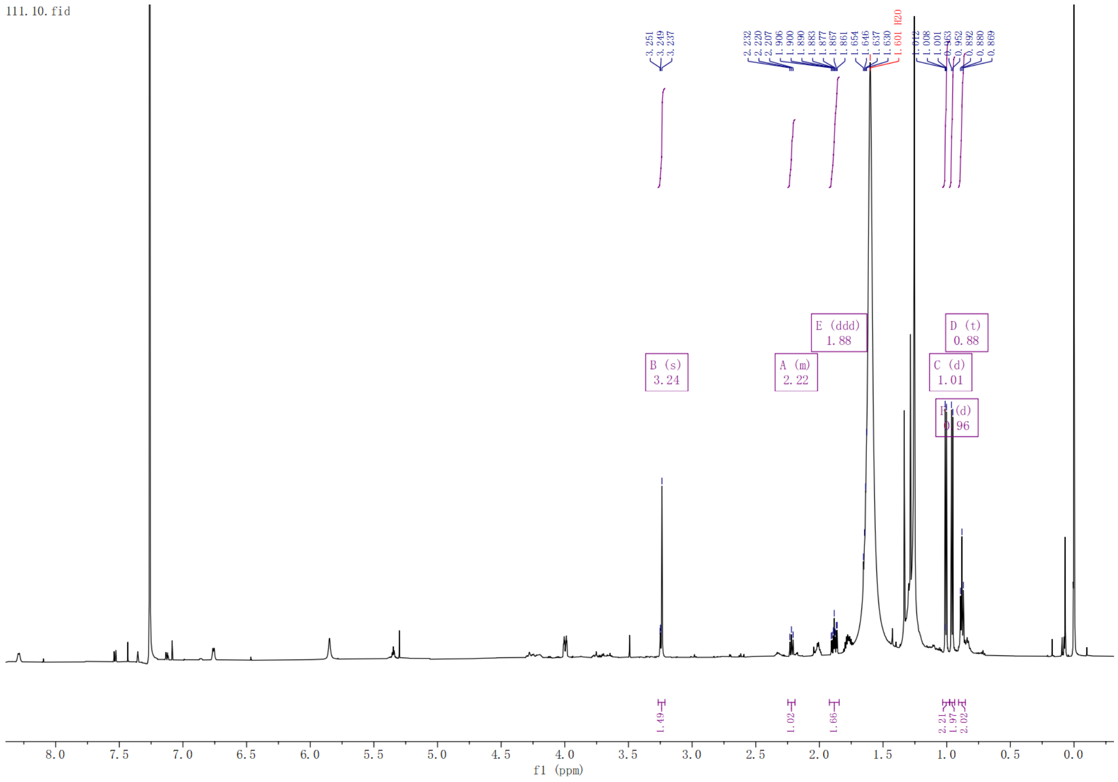

Figure S9. ^{13}C NMR spectrum (151 MHz, $CDCl_3$) of compound **2**.

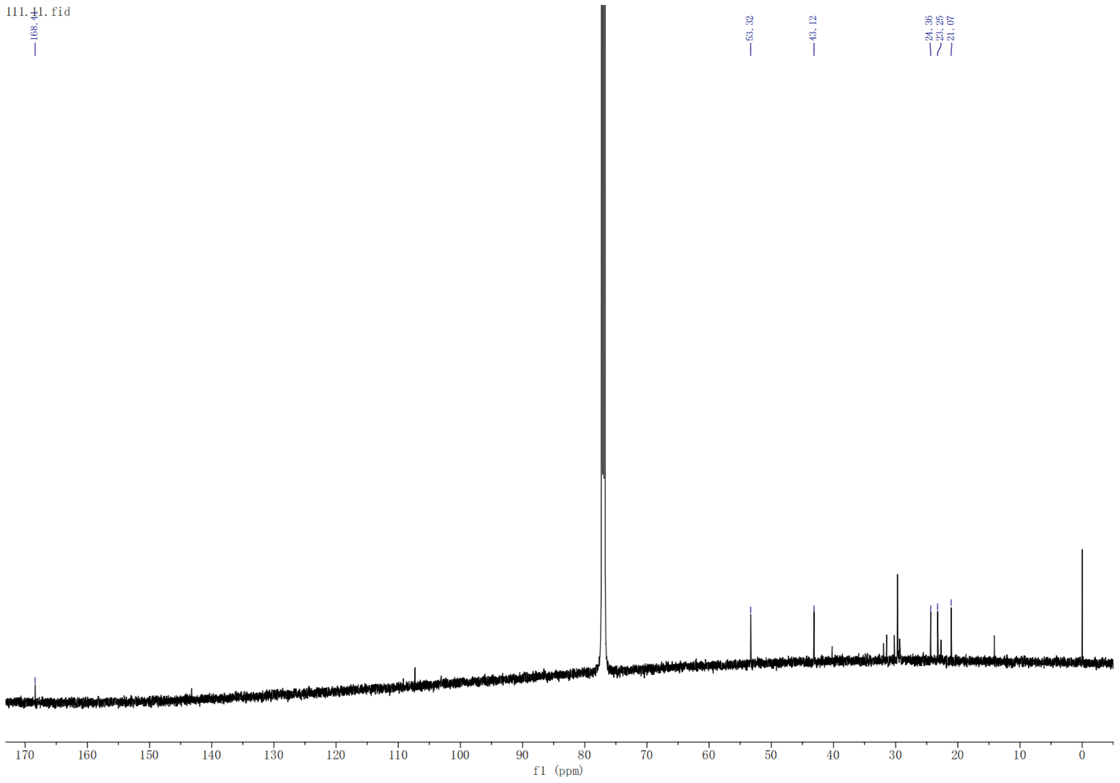

Figure S10. HR-ESI-MS of compound 3

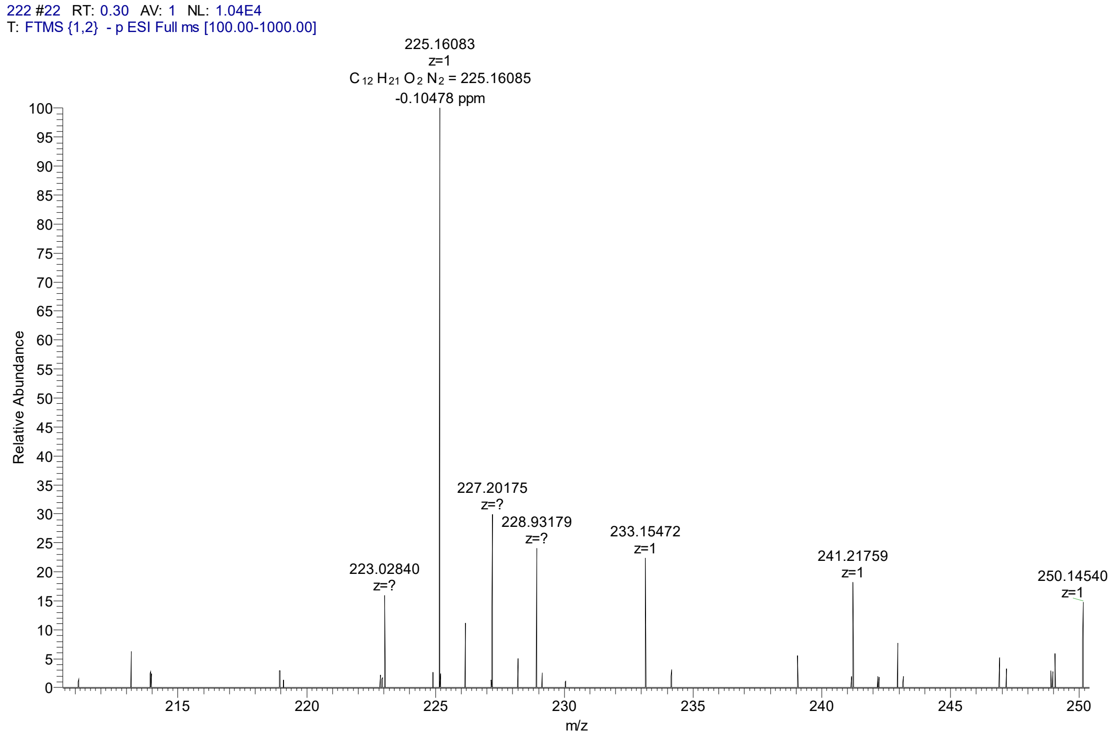

Figure S11.^{1}H NMR spectrum (600 MHz, MeOD) of compound 3.

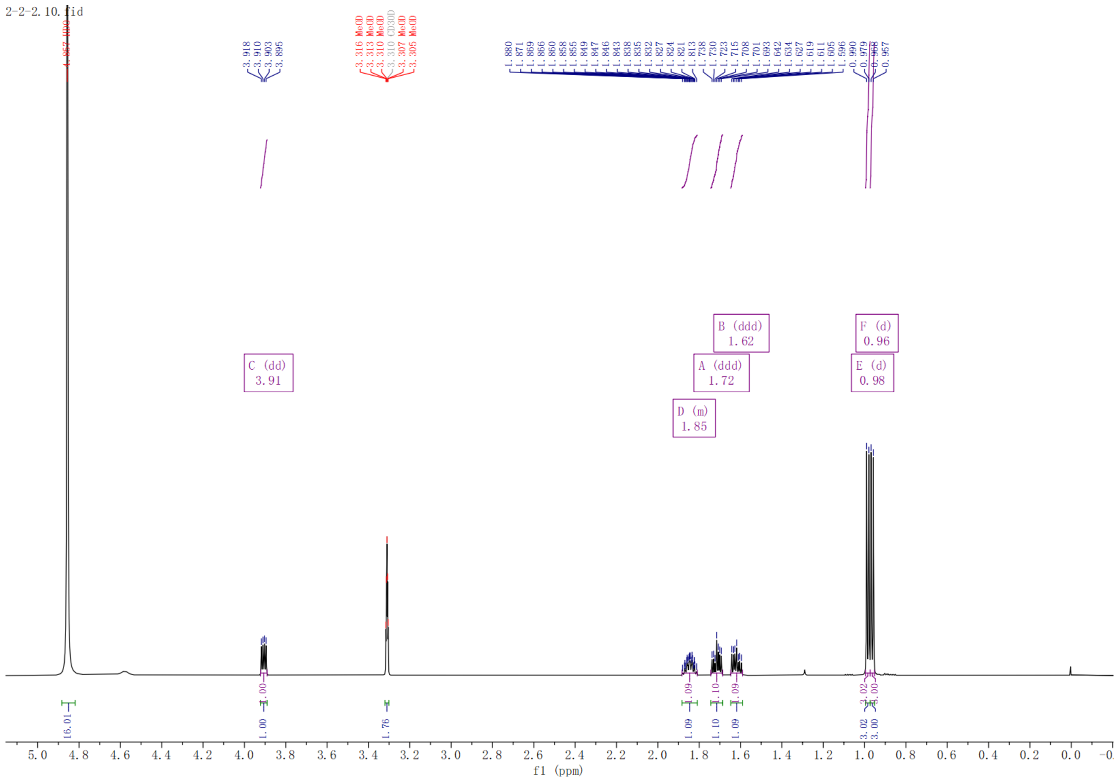

Figure S12. ^{13}C NMR and DEPT spectra (151 MHz, MeOD) of compound **3**.

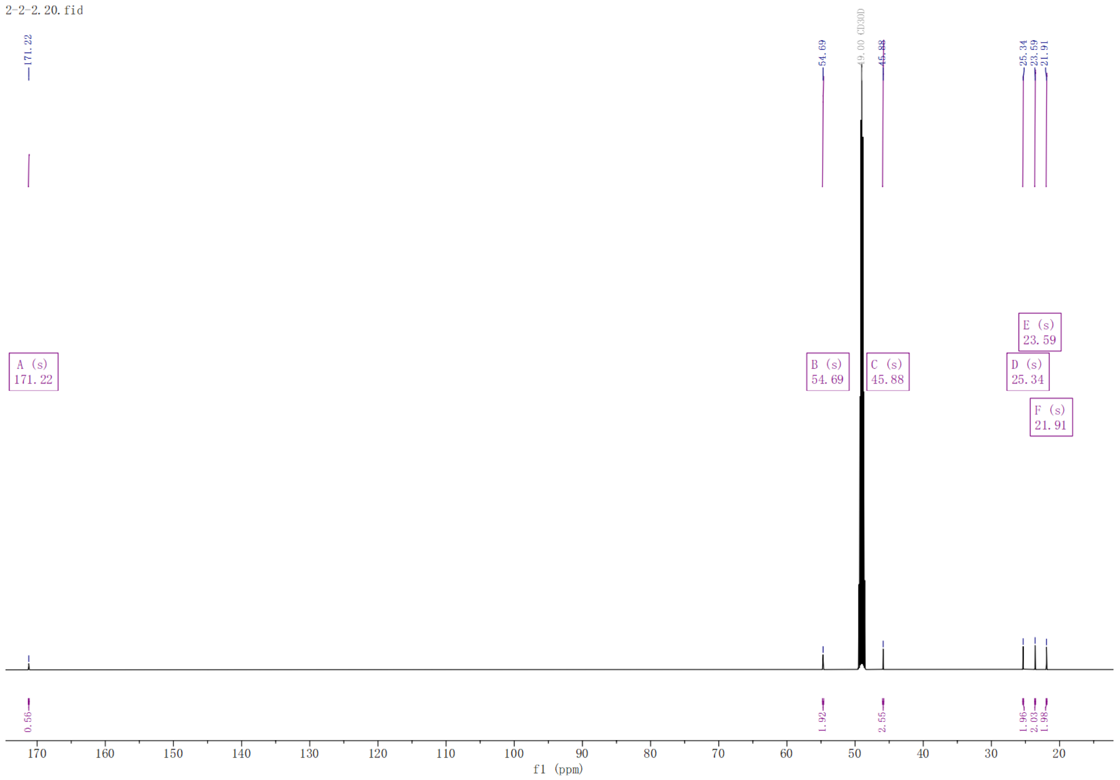

Figure S13. HR-ESI-MS of compound **4**

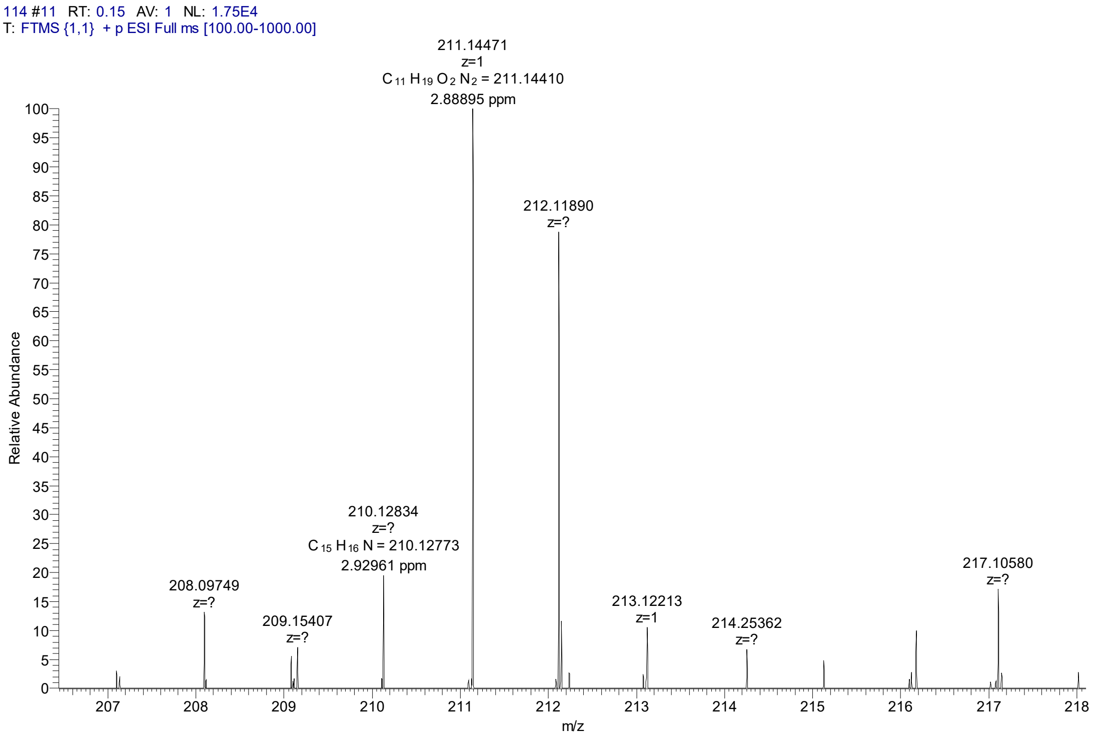

Figure S14.^{1}H NMR spectrum (600 MHz, $CDCl_3$) of compound **4**.

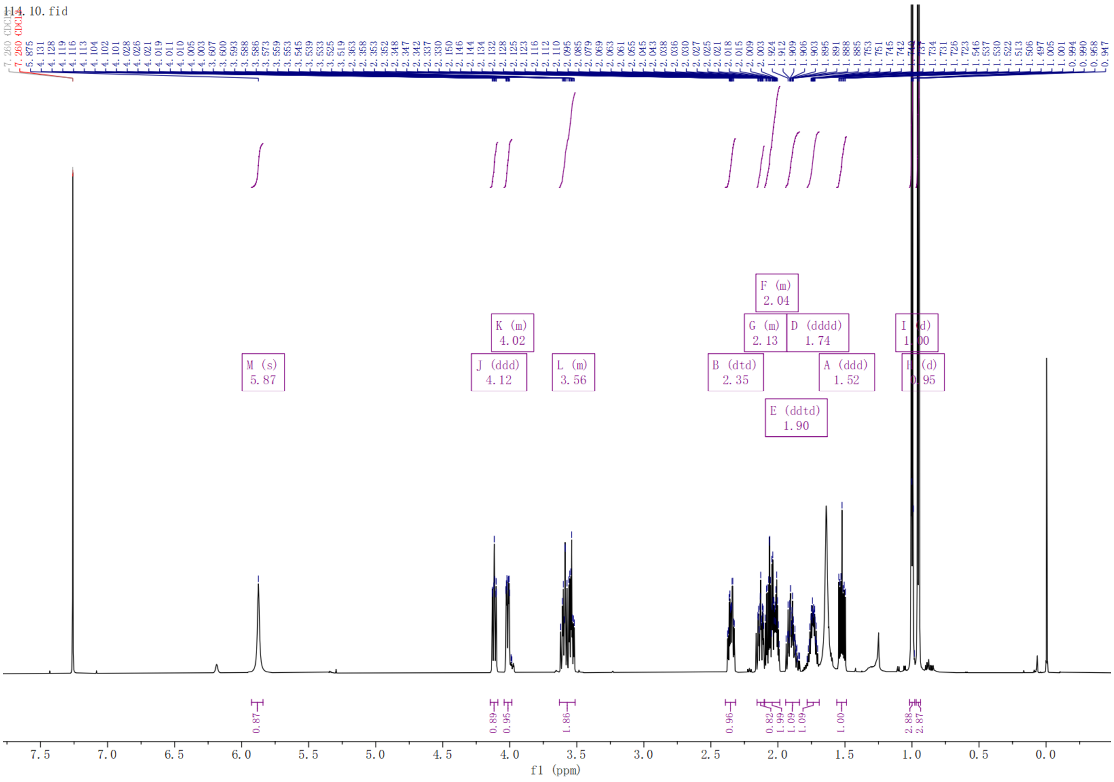

Figure S15.^{13}C NMR spectrum (151 MHz, $CDCl_3$) of compound **4**.

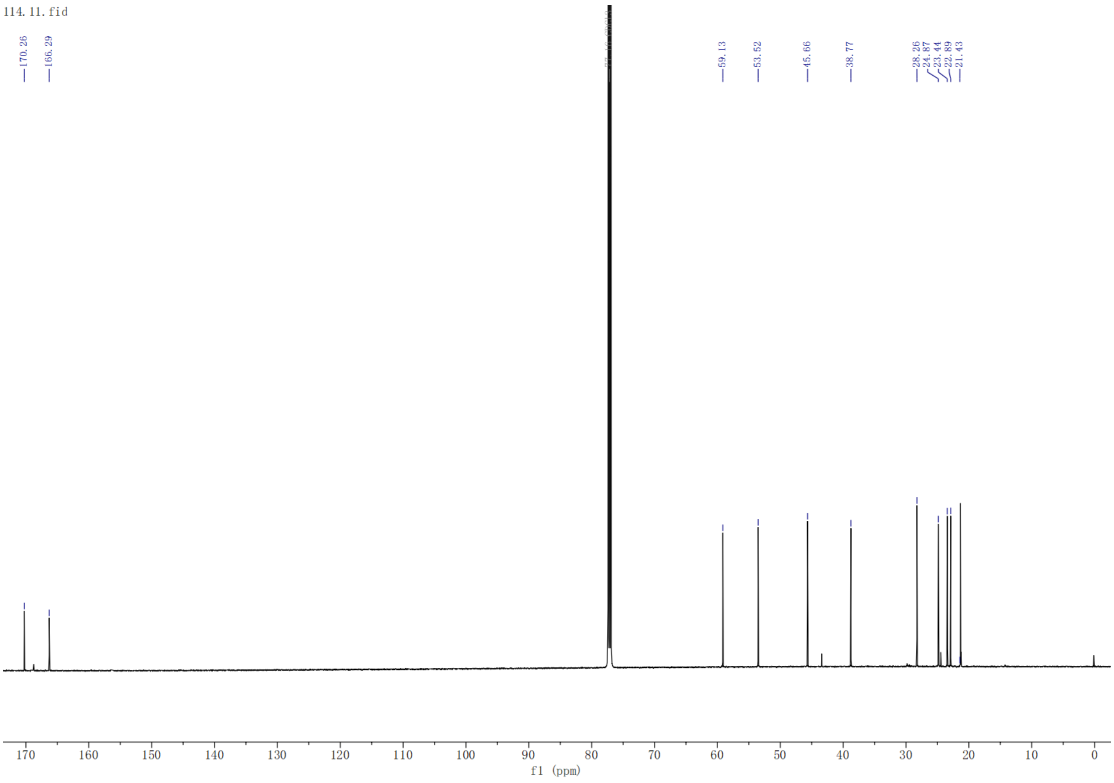

Figure S16. HR-ESI-MS of compound 5

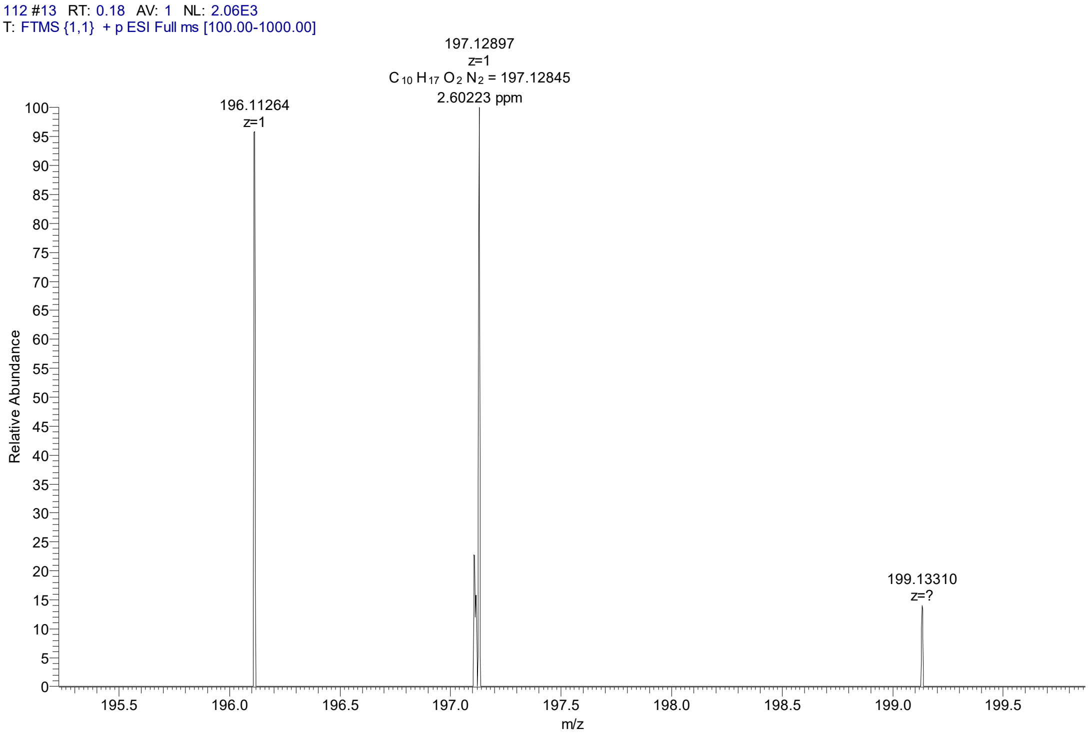

Figure S17.^{1}H NMR spectrum (600 MHz, MeOD) of compound 5.

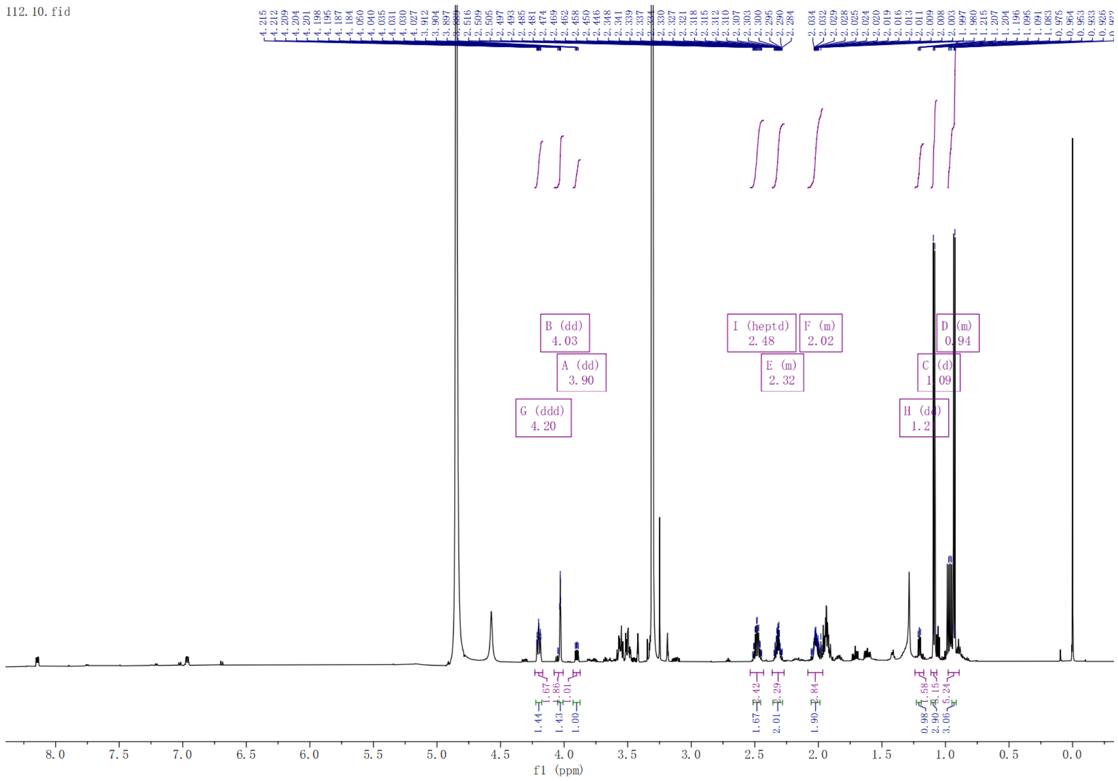

Figure S18.^{13}C NMR spectrum (151 MHz, MeOD) of compound **5**.

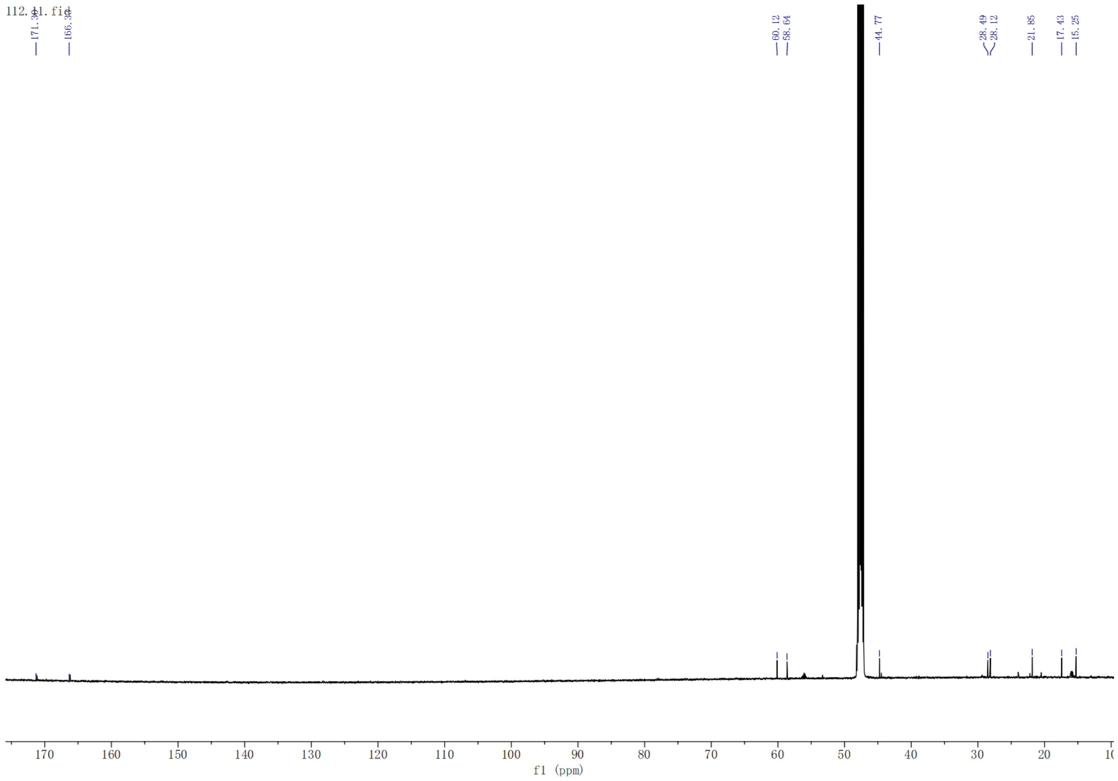

Figure S19. HR-ESI-MS of compound **6**

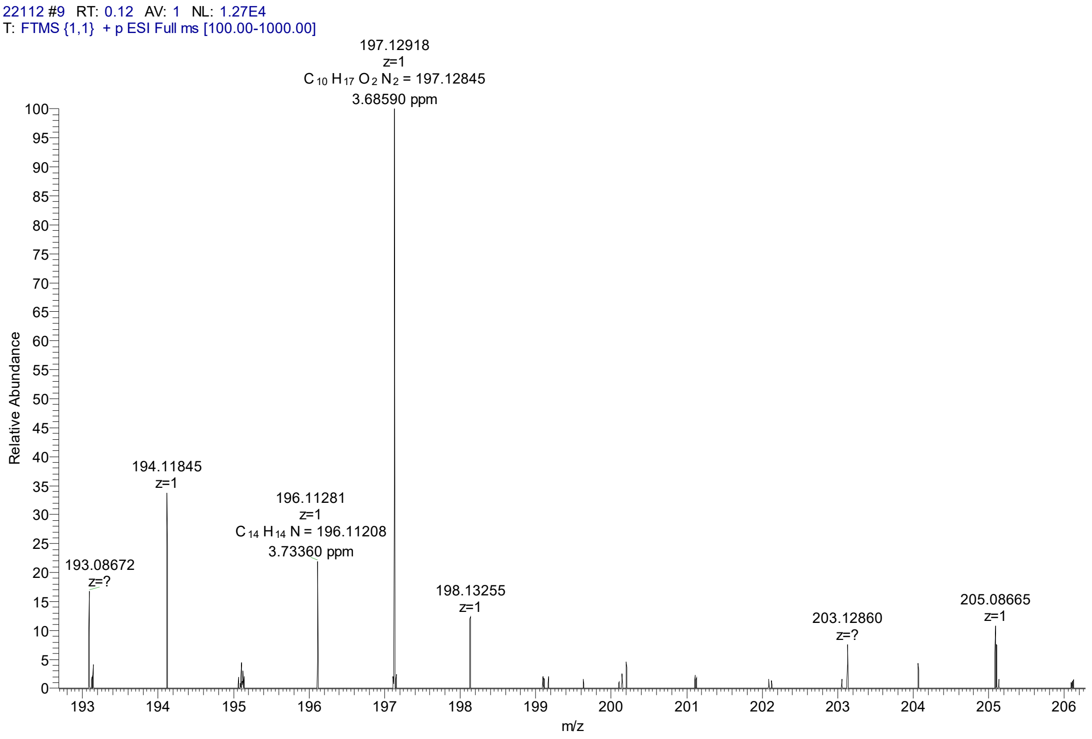

Figure S20.^{1}H NMR spectrum (600 MHz, MeOD) of compound 6.

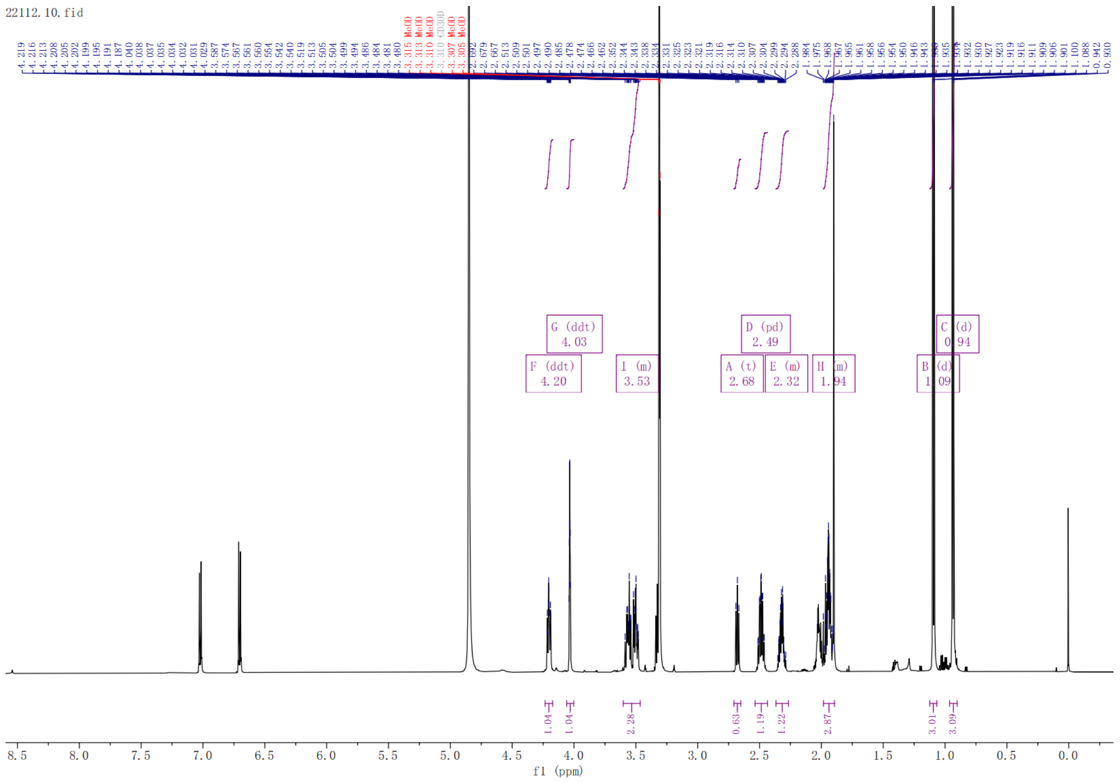

Figure S21.^{13}C NMR and DEPT spectra (151 MHz, MeOD) of compound 6.

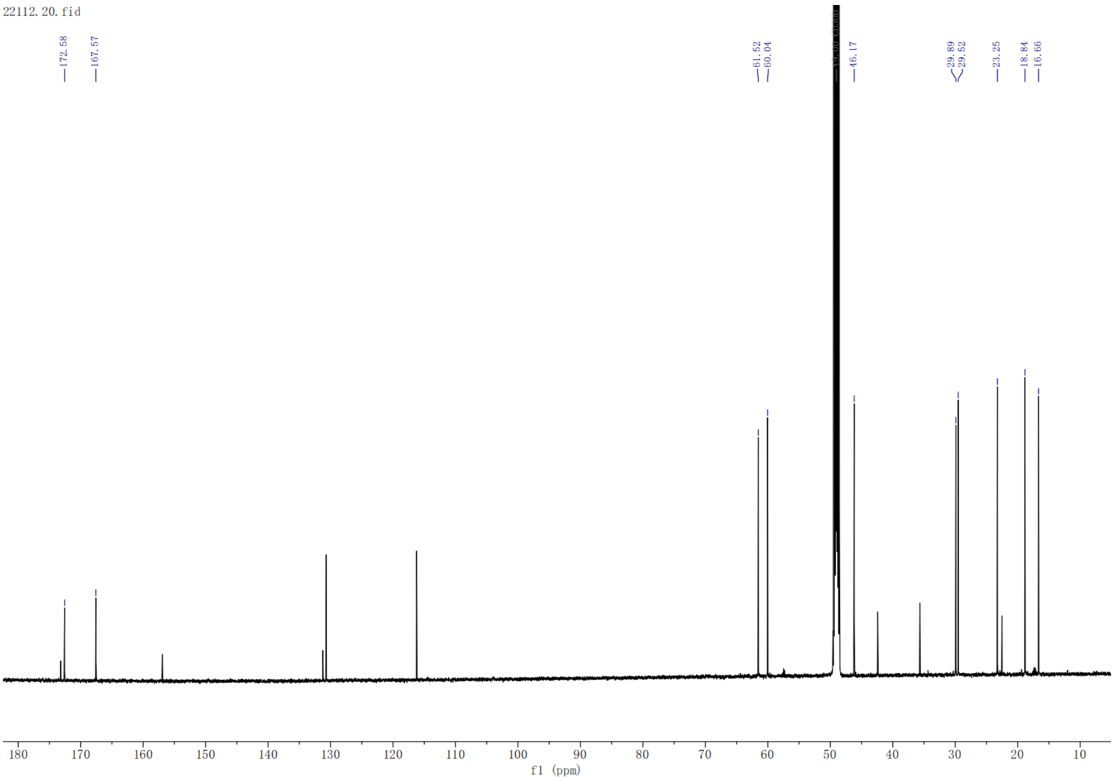

Figure S22. HR-ESI-MS of compound **7**

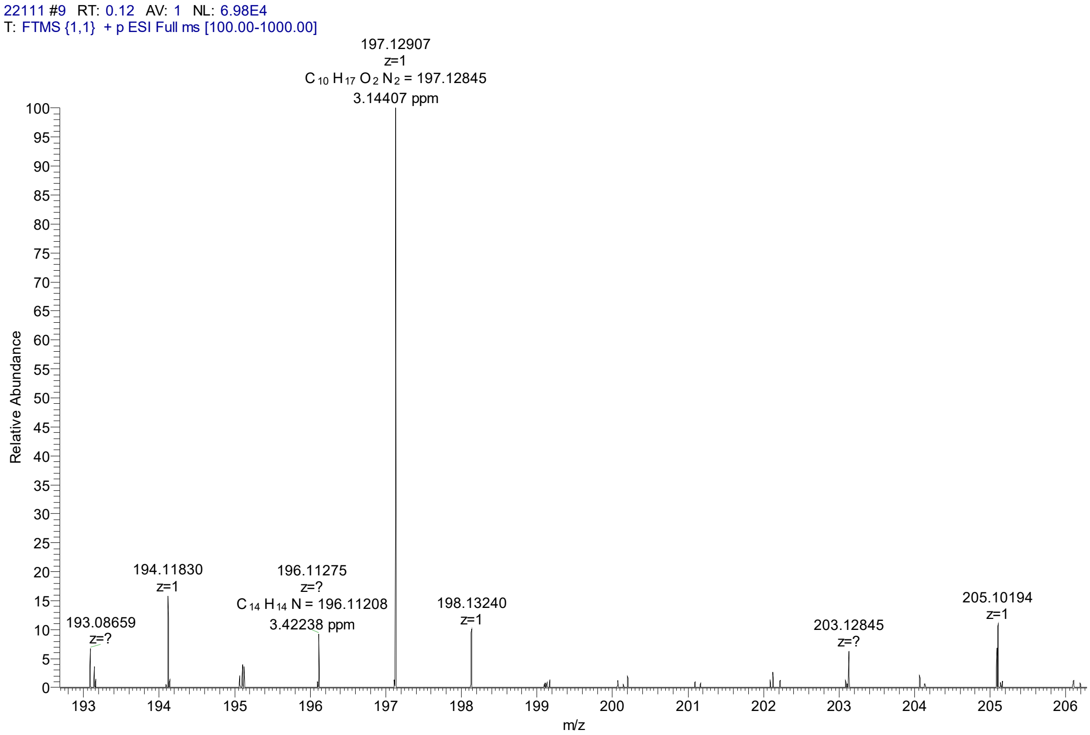

Figure S23.^{1}H NMR spectrum (600 MHz, MeOD) of compound **7**.

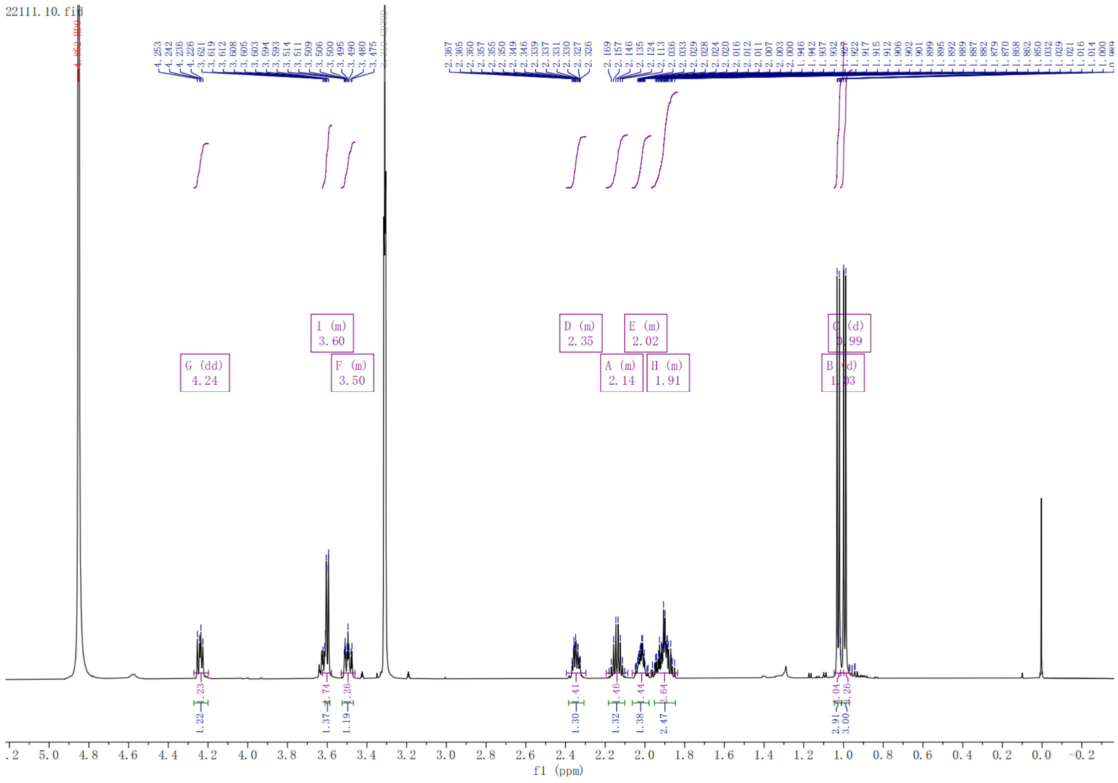

Figure S24.^{13}C NMR and DEPT spectra (151 MHz, MeOD) of compound **7**.

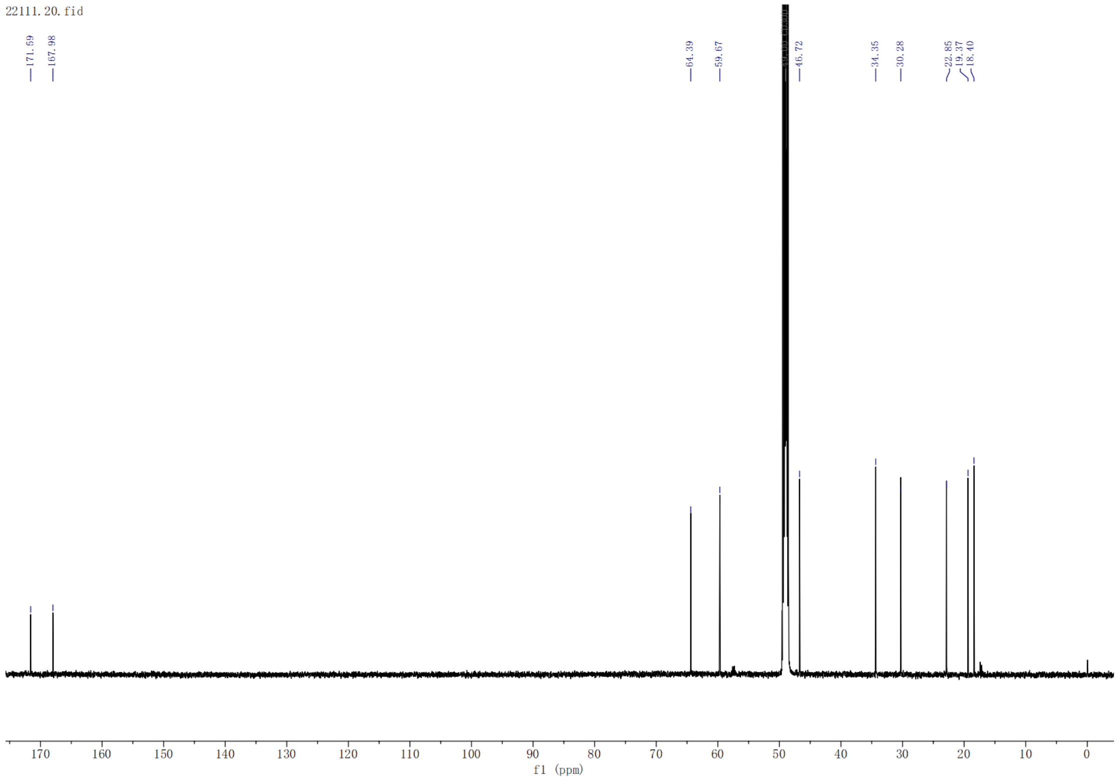

Figure S25. HR-ESI-MS of compound **8**

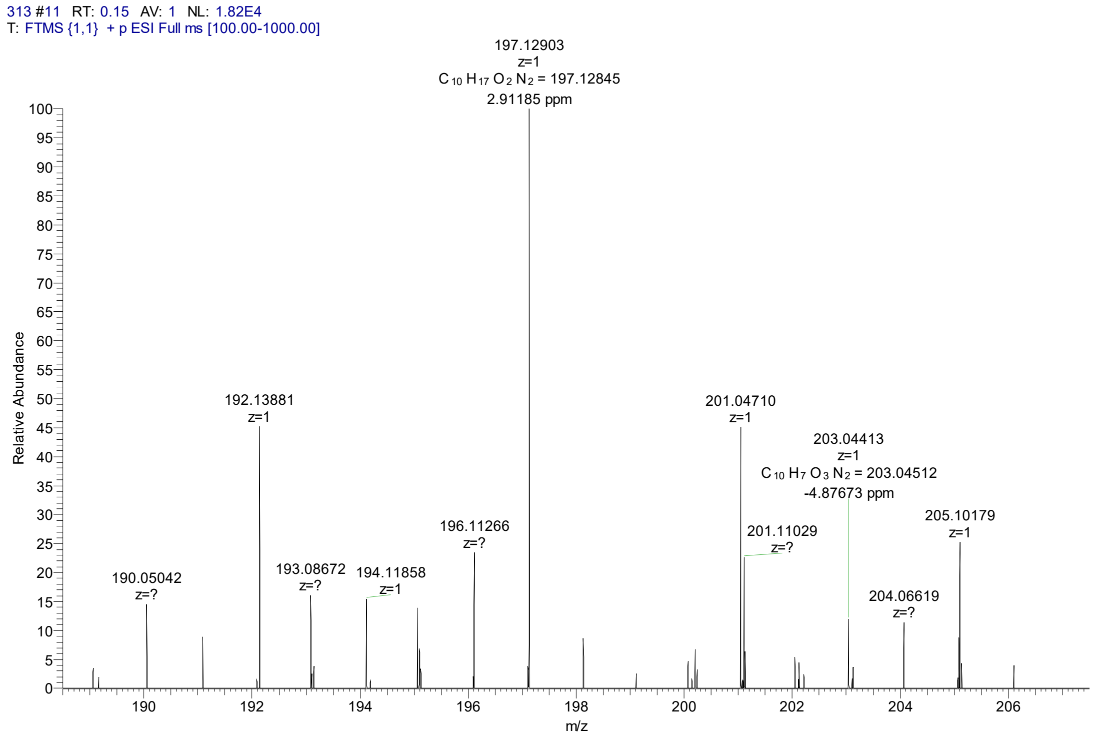

Figure S26.^{1}H NMR spectrum (600 MHz, MeOD) of compound **8**.

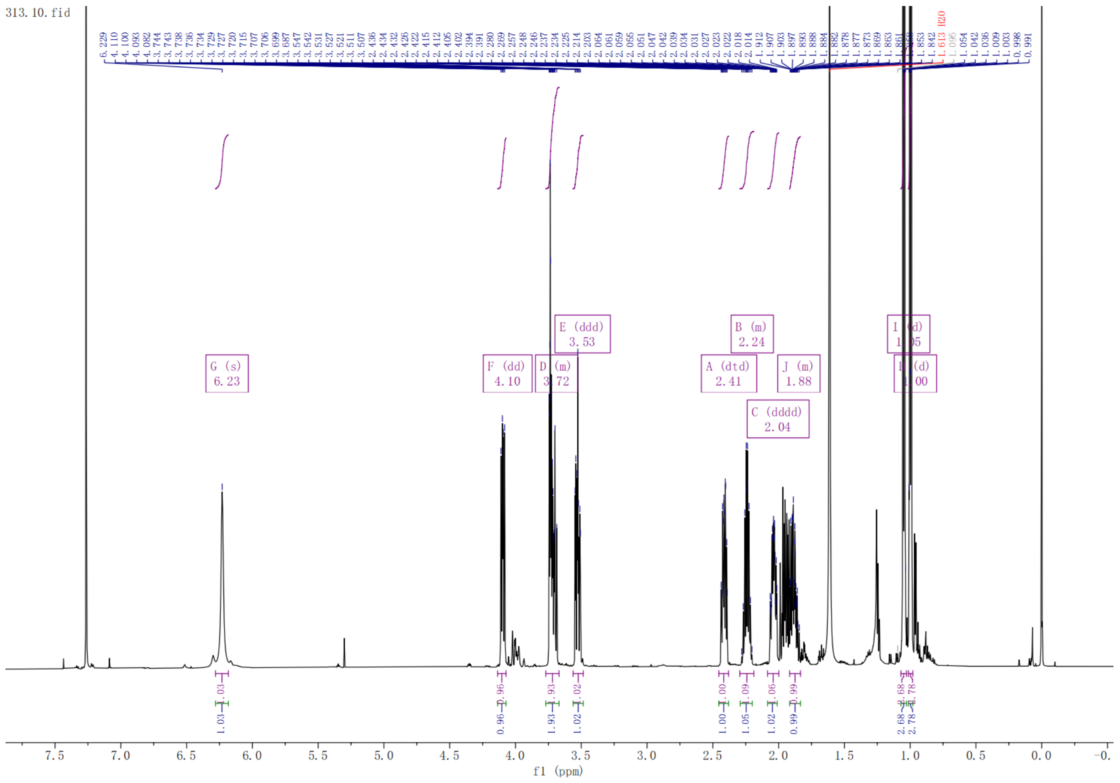

Figure S27.^{13}C NMR and DEPT spectra (151 MHz, MeOD) of compound **8**.

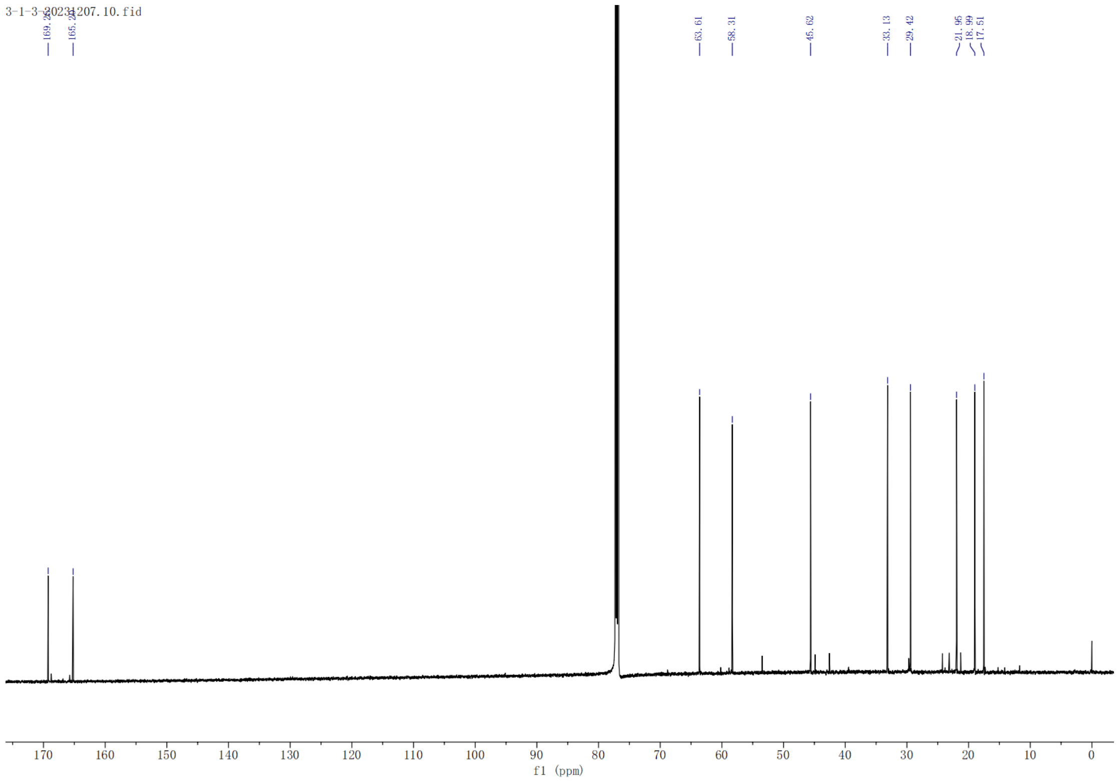

Figure S28. HR-ESI-MS of compound **9**

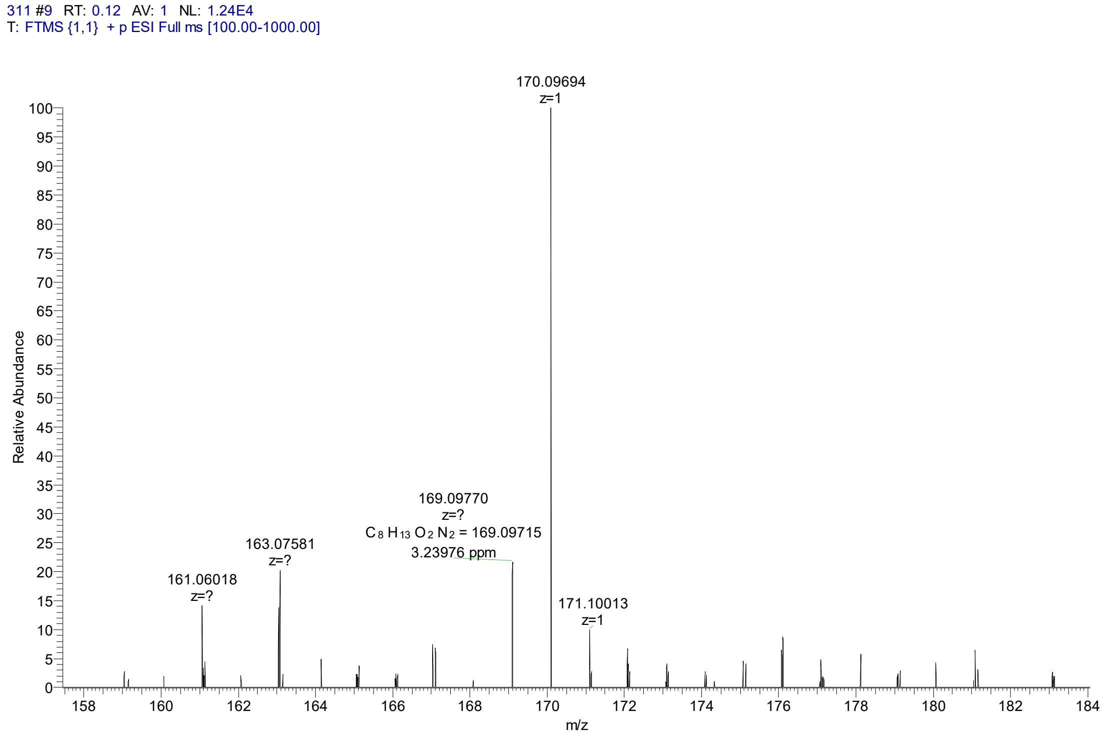

Figure S29.^{1}H NMR spectrum (600 MHz, MeOD) of compound **9**

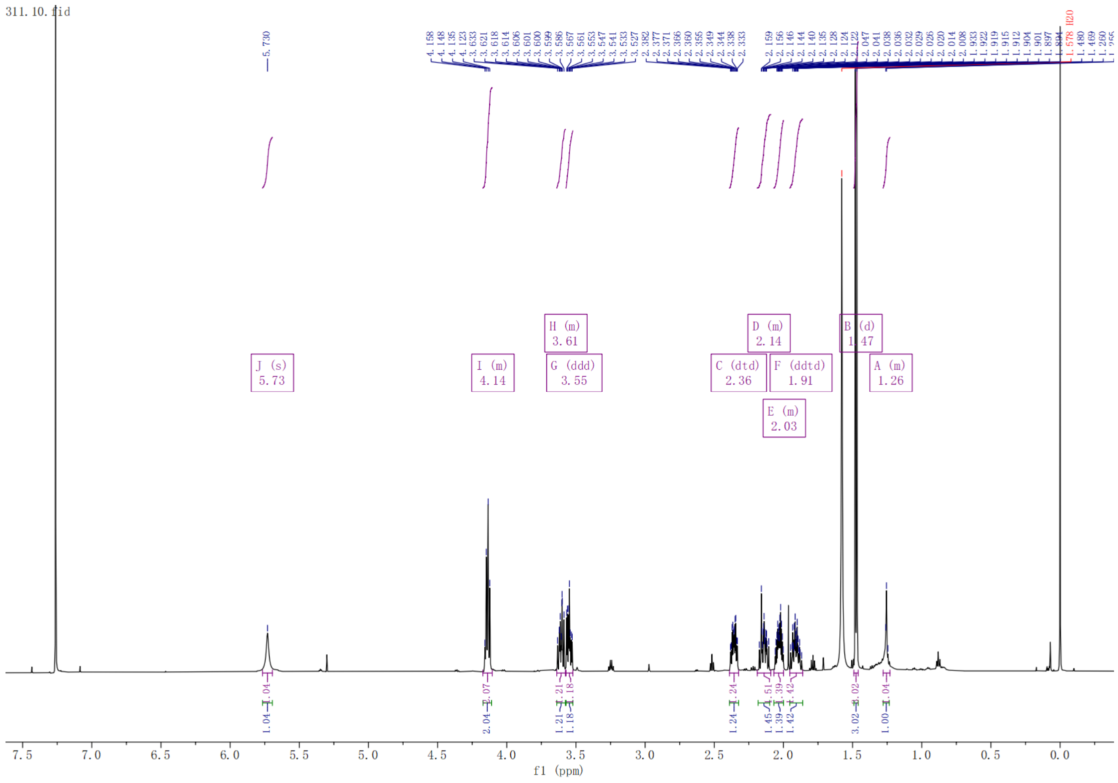

Figure S30.^{13}C NMR and DEPT spectra (151 MHz, MeOD) of compound **9**.

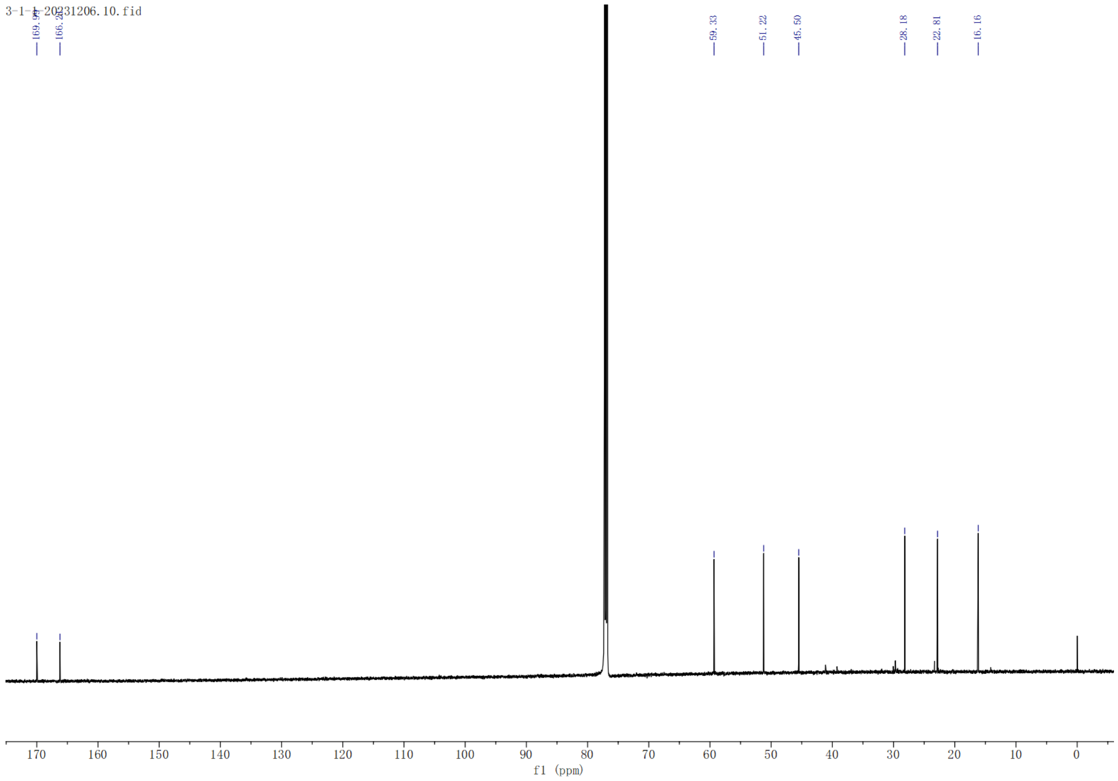

Figure S31. HR-ESI-MS of compound **10**

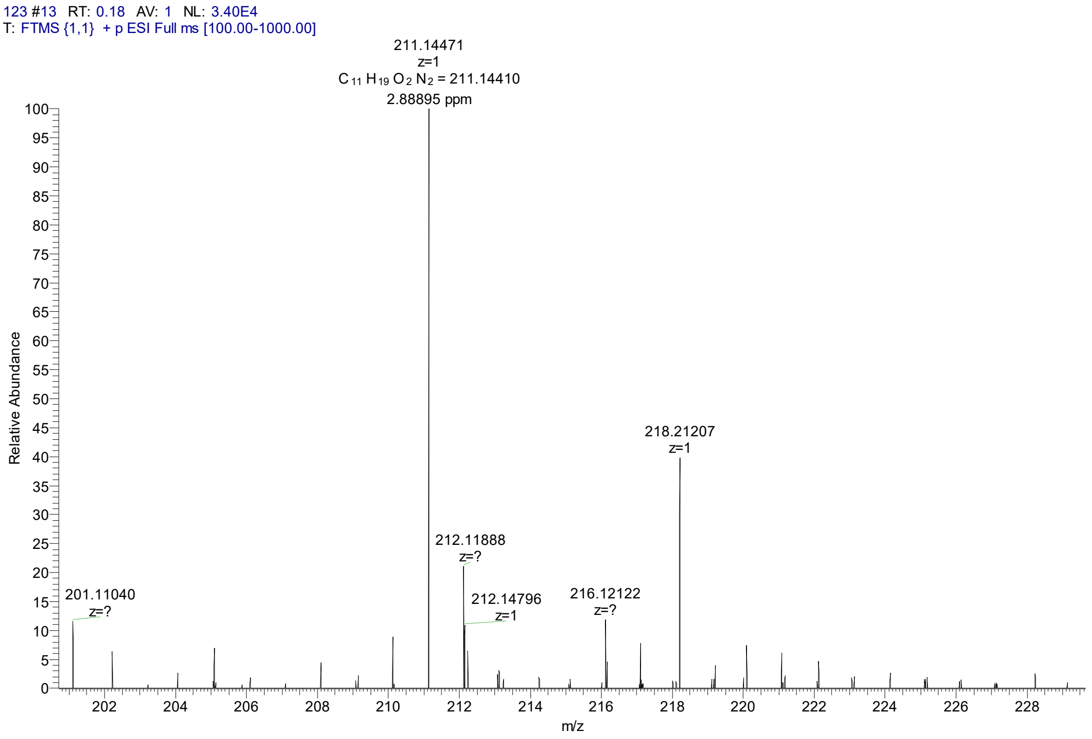

Figure S32.^{1}H NMR spectrum (600 MHz, MeOD) of compound **10**.

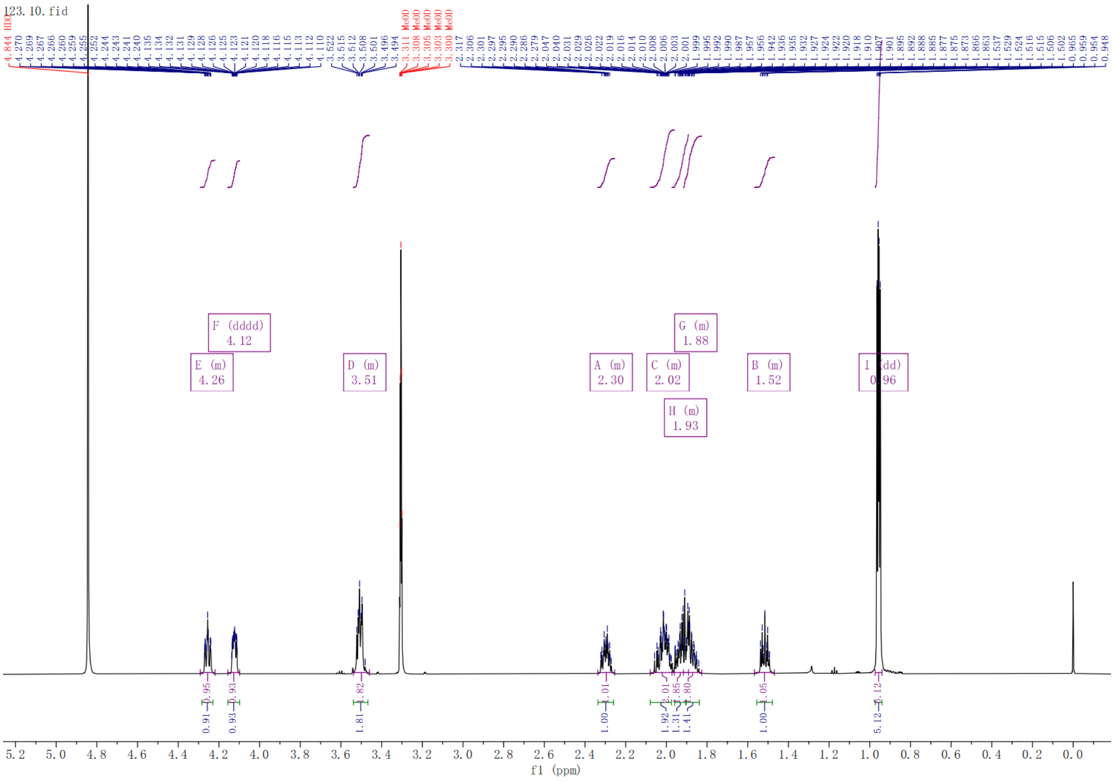

Figure S33.^{13}C NMR and DEPT spectra (151 MHz, MeOD) of compound **10**.

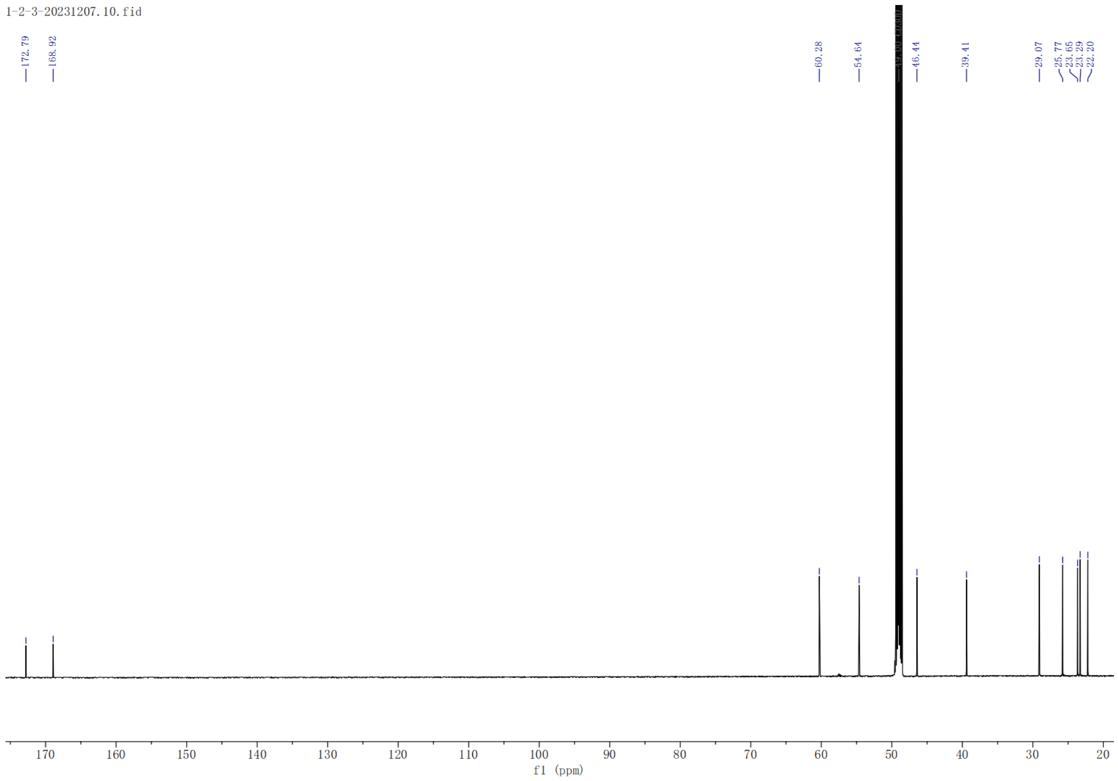

Figure S34.^{1}H NMR spectrum (600 MHz, MeOD) of compound **11**.

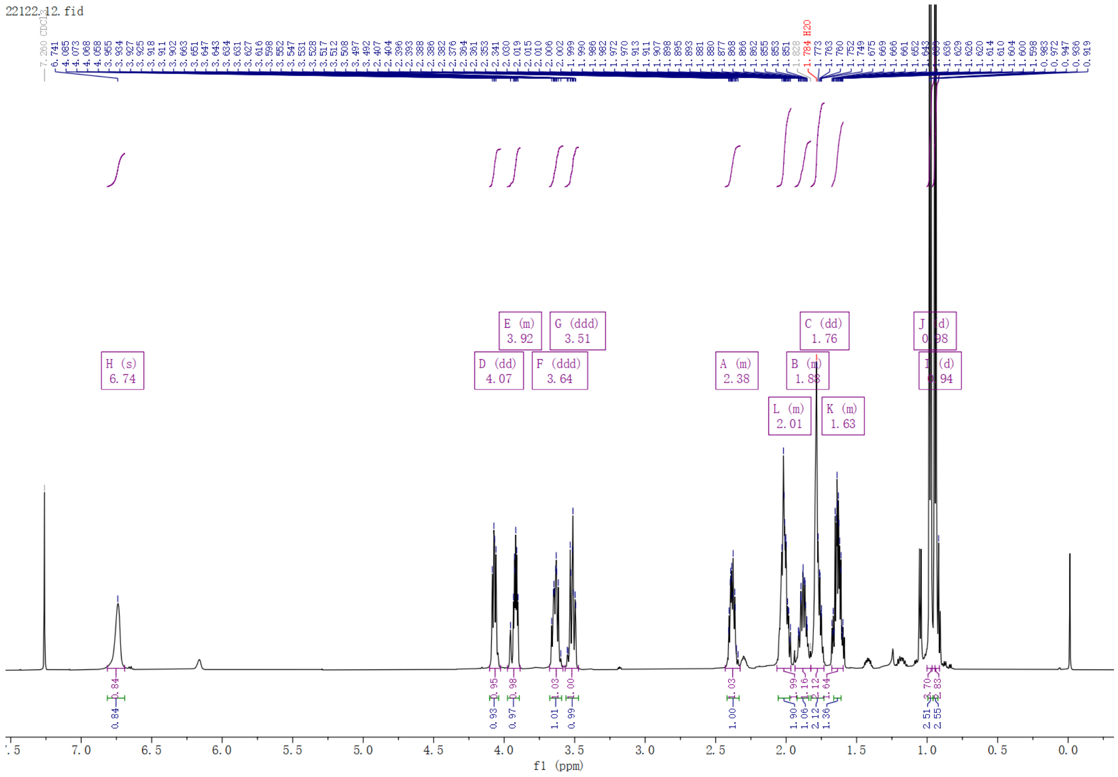

Figure S35.^{13}C NMR and DEPT spectra (151 MHz, MeOD) of compound **11**.

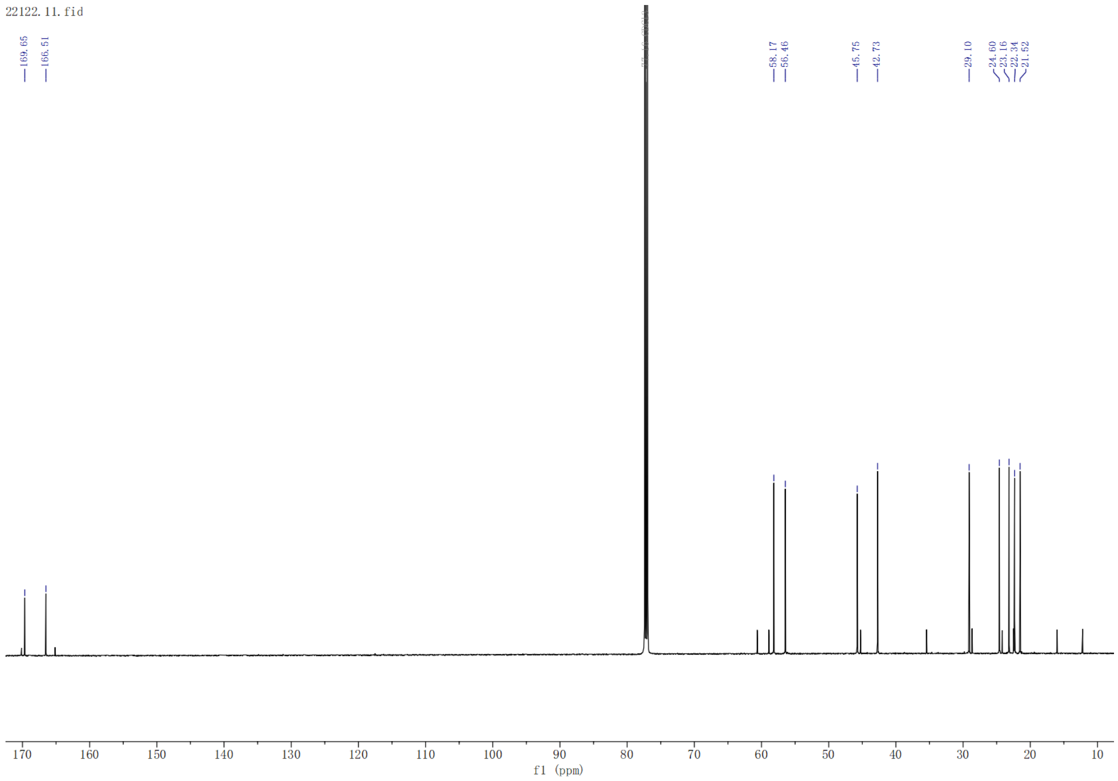

Figure S36. HR-ESI-MS of compound 12

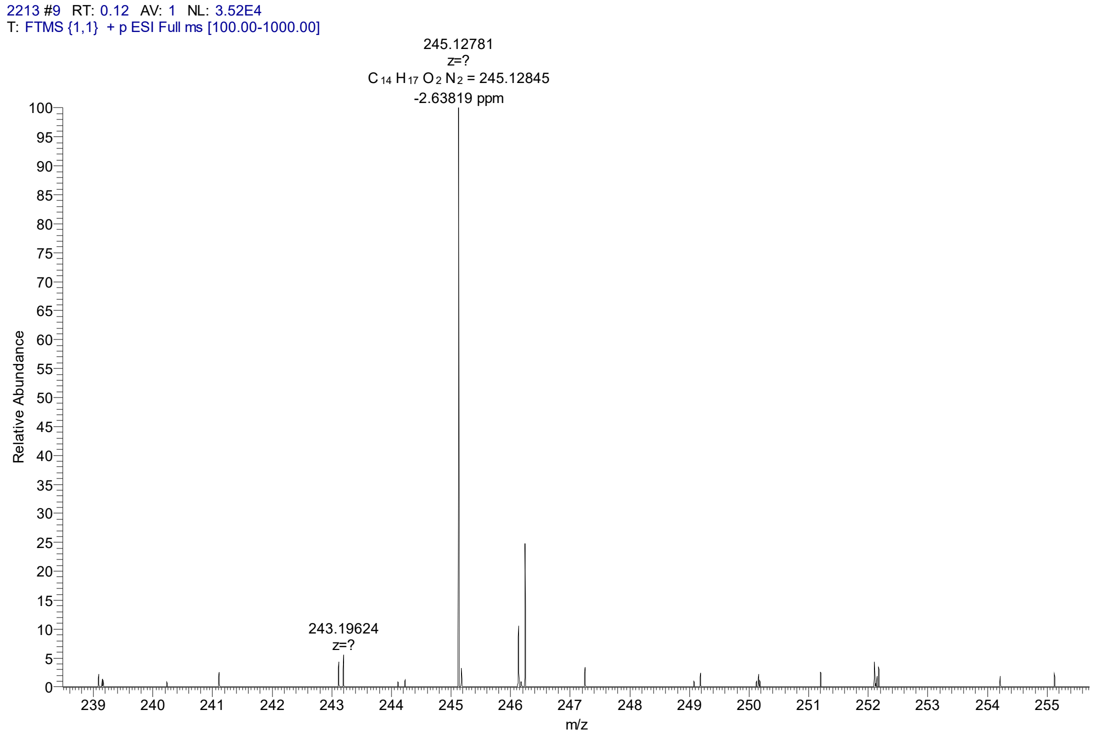

Figure S37.^{1}H NMR spectrum (600 MHz, MeOD) of compound 12.

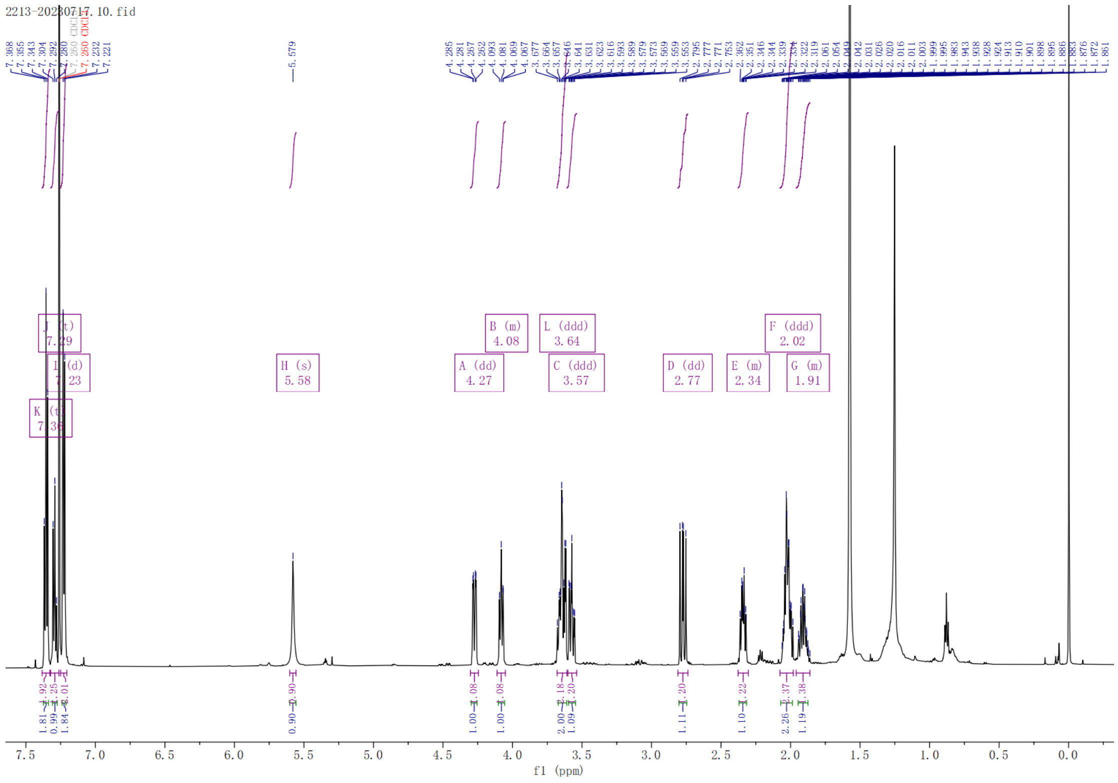

Figure S38.^{13}C NMR and DEPT spectra (151 MHz, MeOD) of compound **12**.

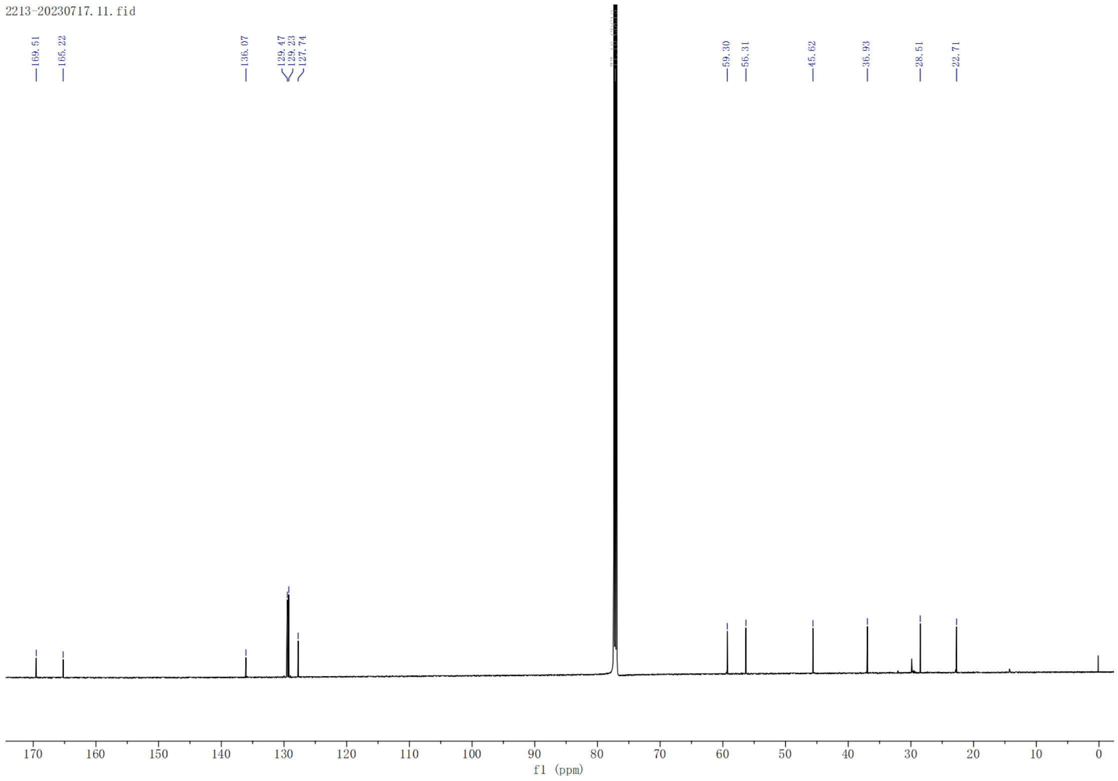

Figure S39. HR-ESI-MS of compound **13**

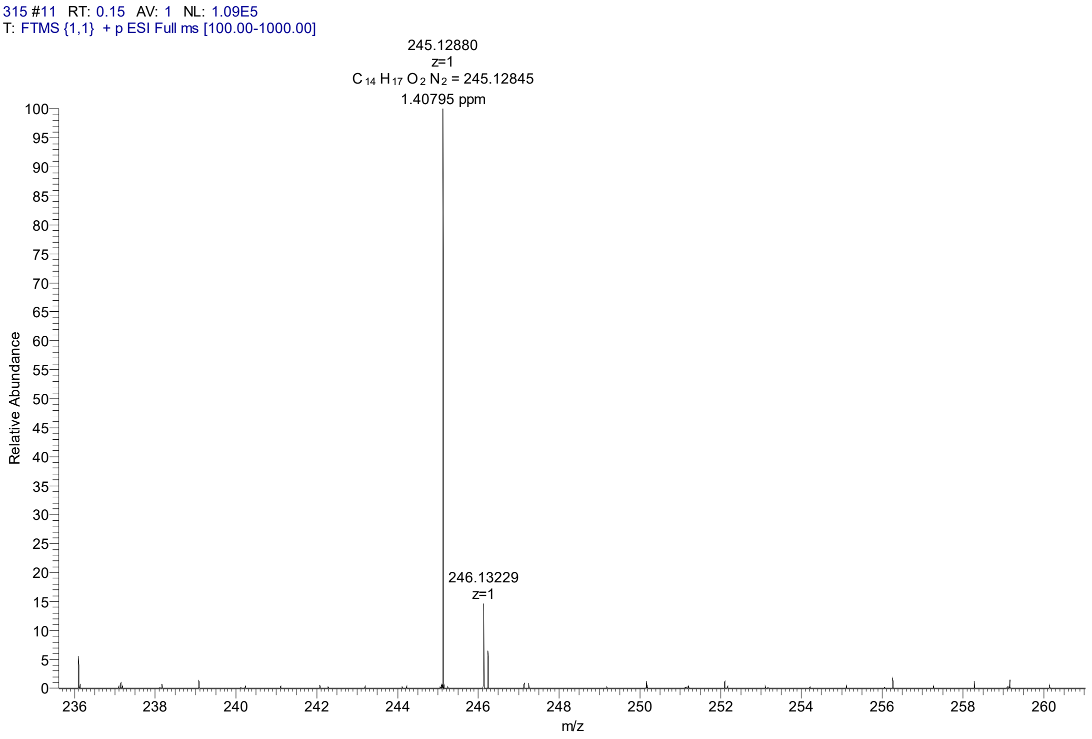

Figure S40.^{1}H NMR spectrum (600 MHz, MeOD) of compound **13**.

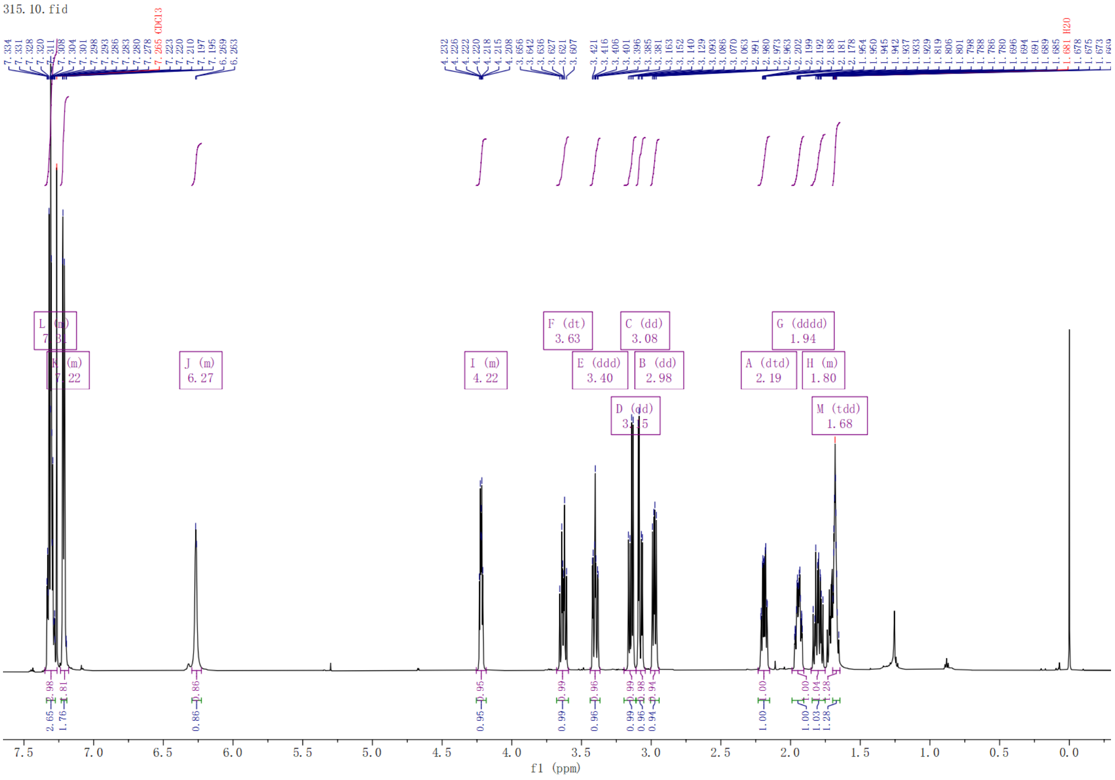

Figure S41.^{13}C NMR and DEPT spectra (151 MHz, MeOD) of compound **13**.

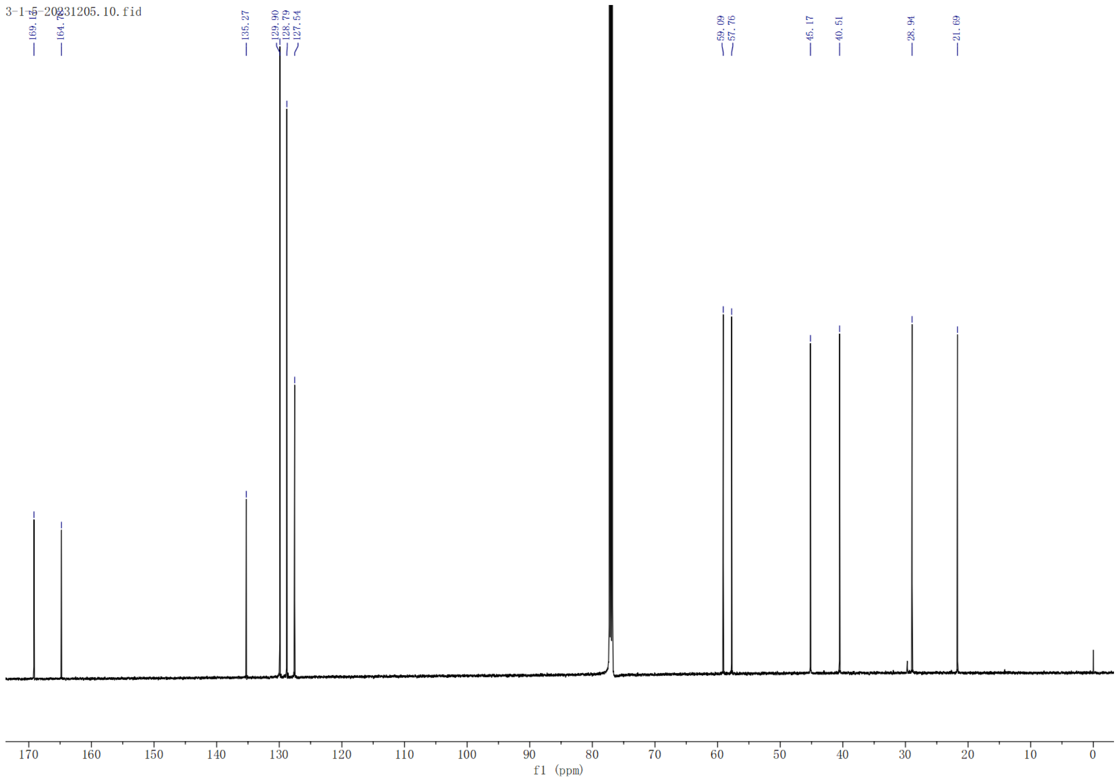

Figure S42. HR-ESI-MS of compound **14**

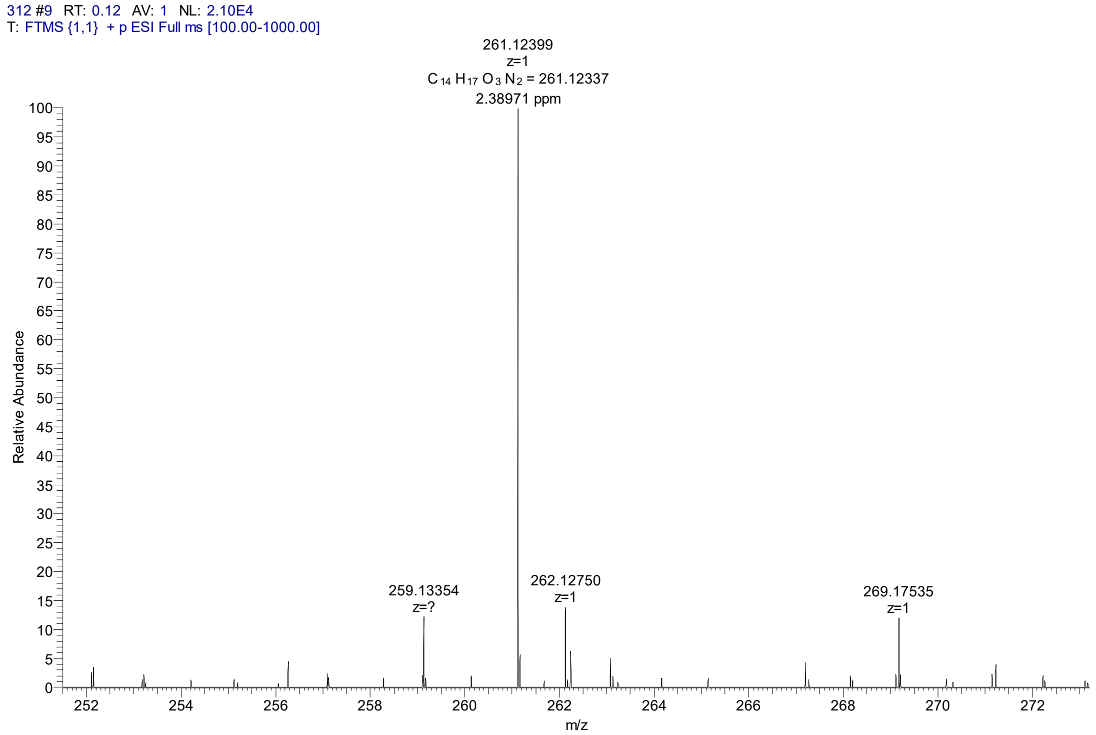

Figure S43.^{1}H NMR spectrum (600 MHz, MeOD) of compound **14**.

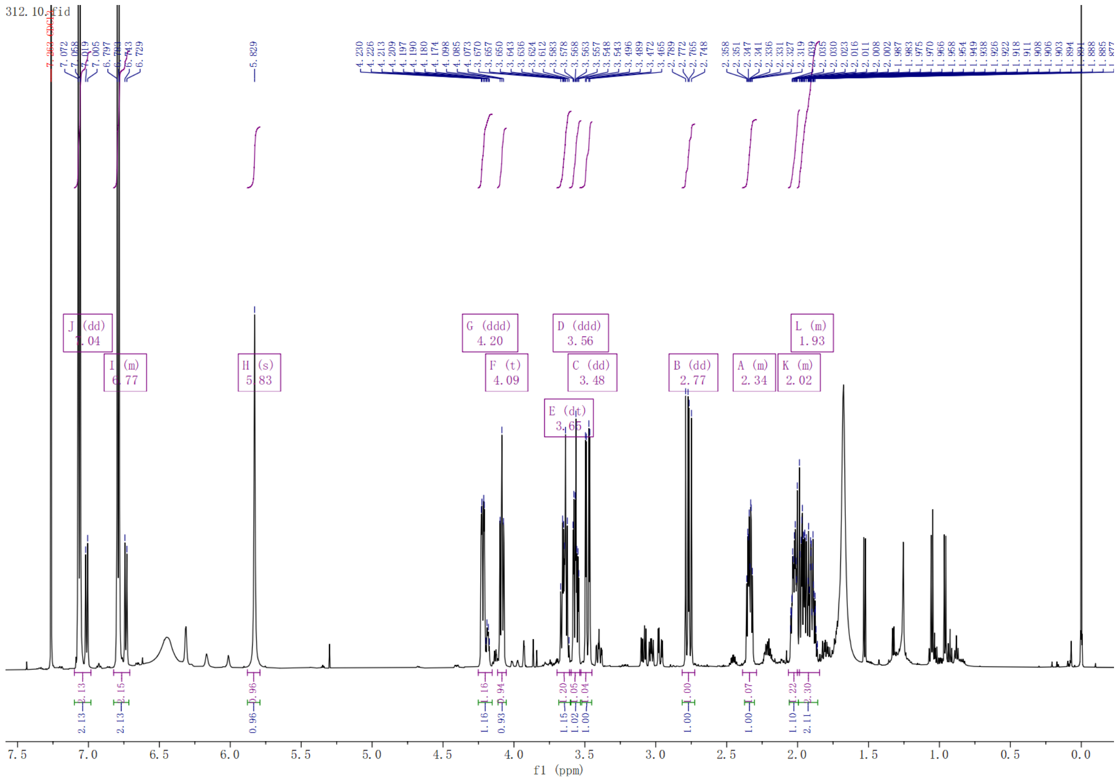

Figure S44.^{13}C NMR and DEPT spectra (151 MHz, MeOD) of compound **14**.

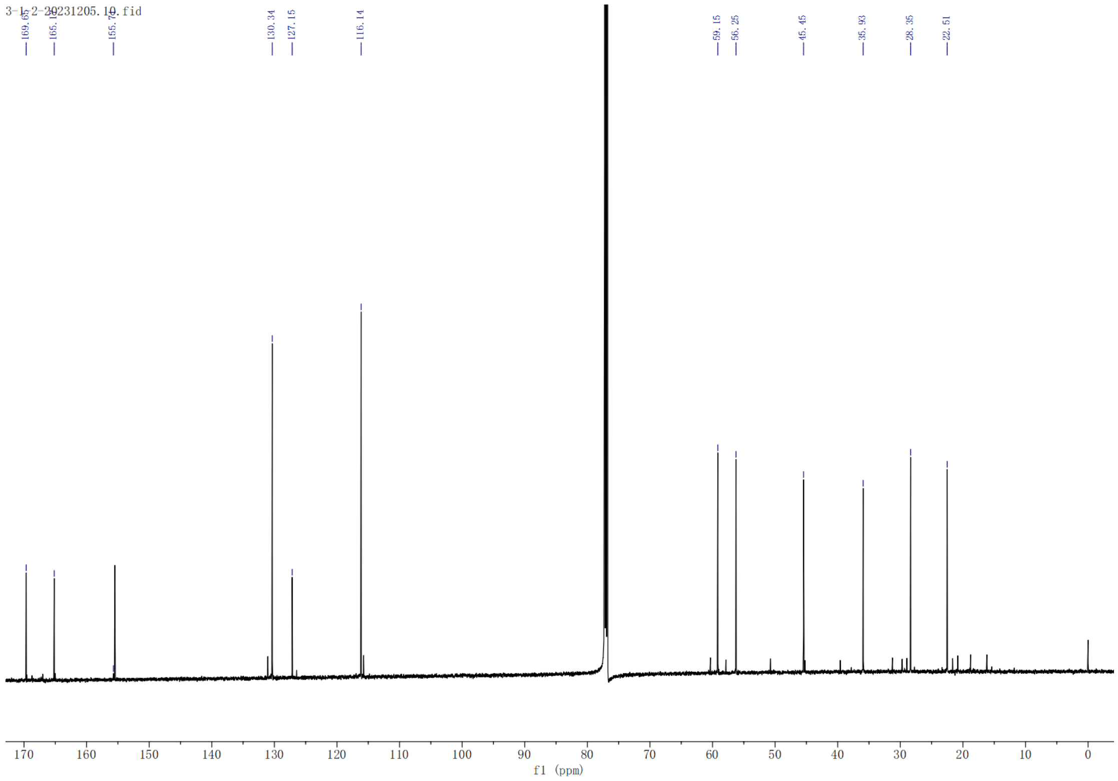

Figure S45. HR-ESI-MS of compound **15**

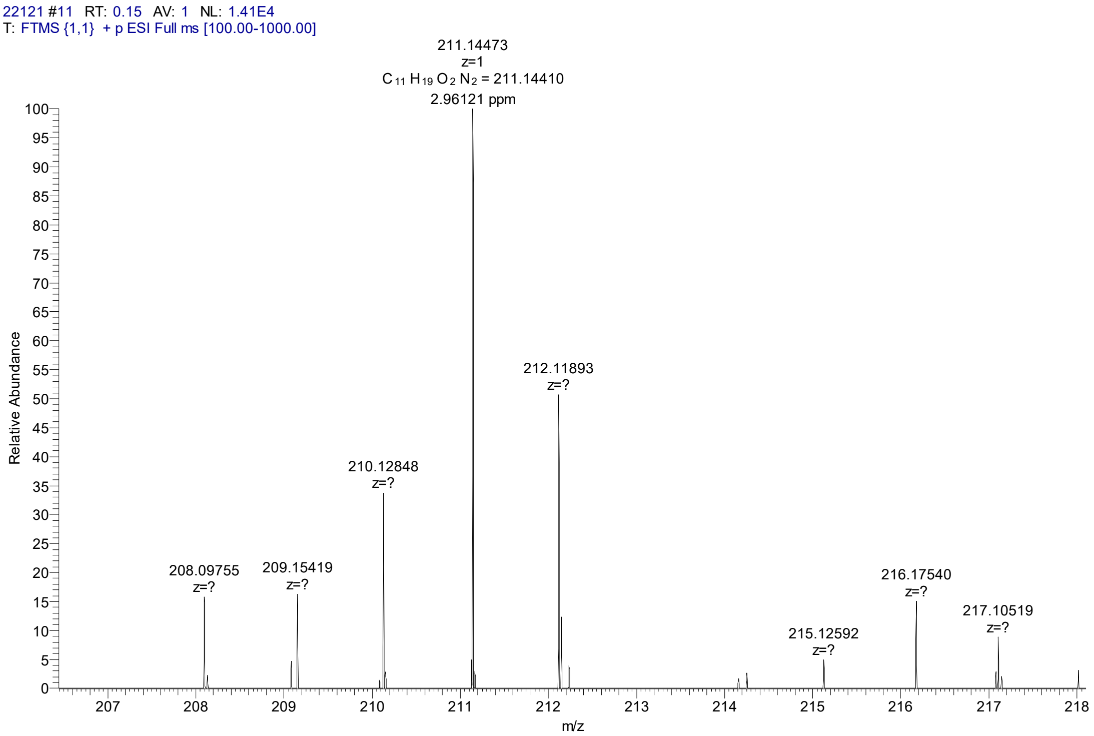

Figure S46.^{1}H NMR spectrum (600 MHz, MeOD) of compound **15**.

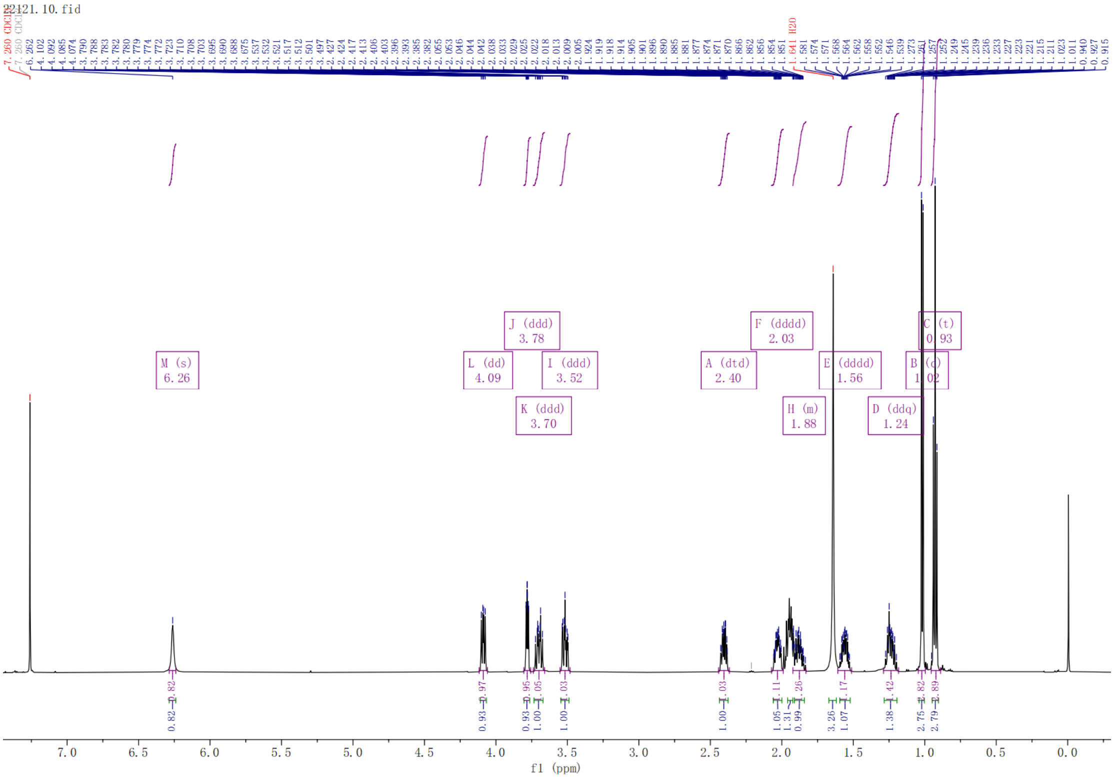

Figure S47.^{13}C NMR and DEPT spectra (151 MHz, MeOD) of compound **15**.

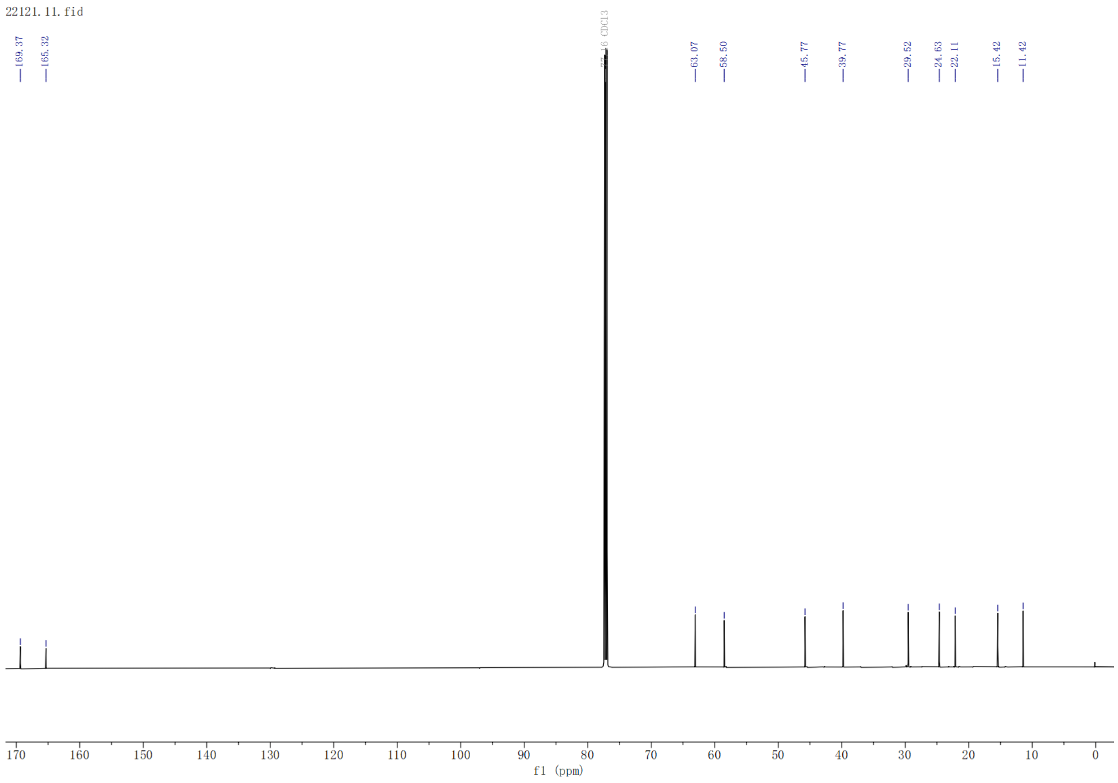

Figure S48. HR-ESI-MS of compound **16**

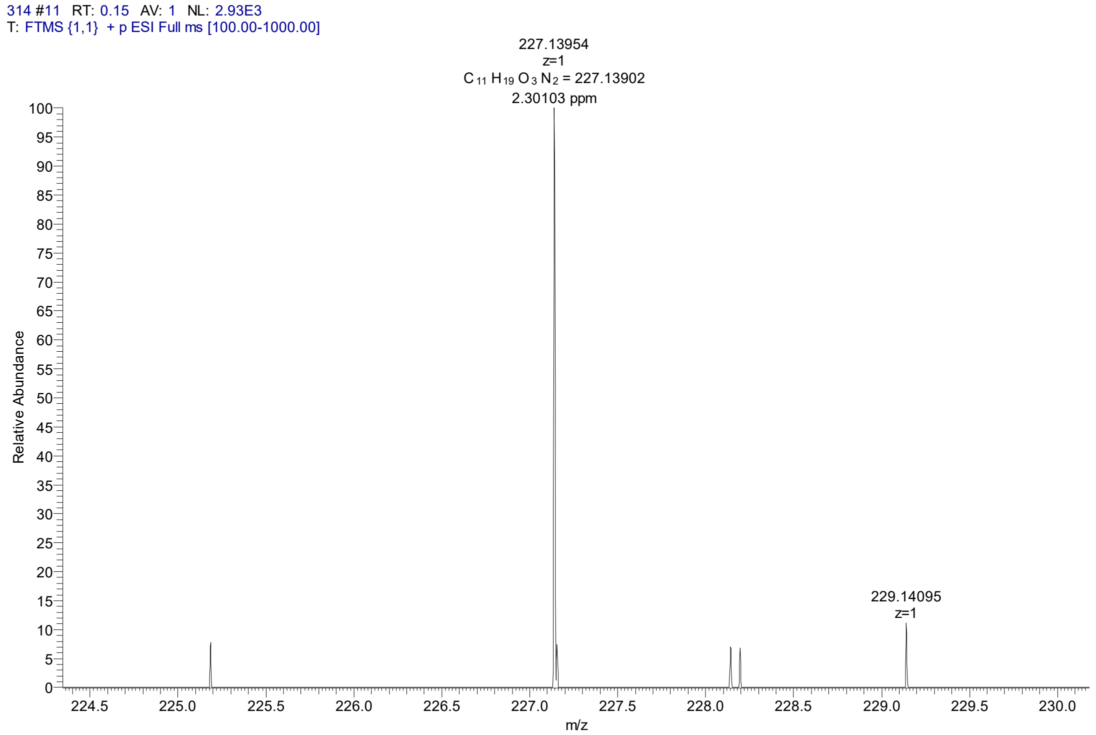

Figure S49.^{1}H NMR spectrum (600 MHz, MeOD) of compound **16**.

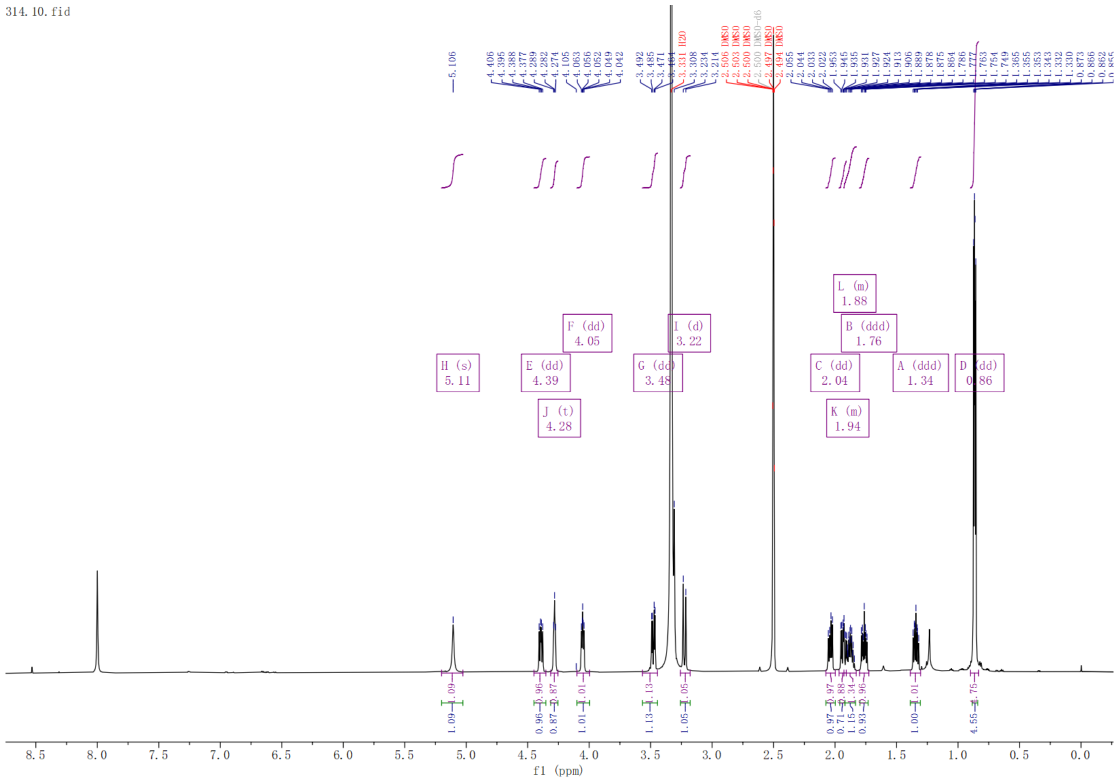

Figure S50.^{13}C NMR and DEPT spectra (151 MHz, MeOD) of compound **16**.

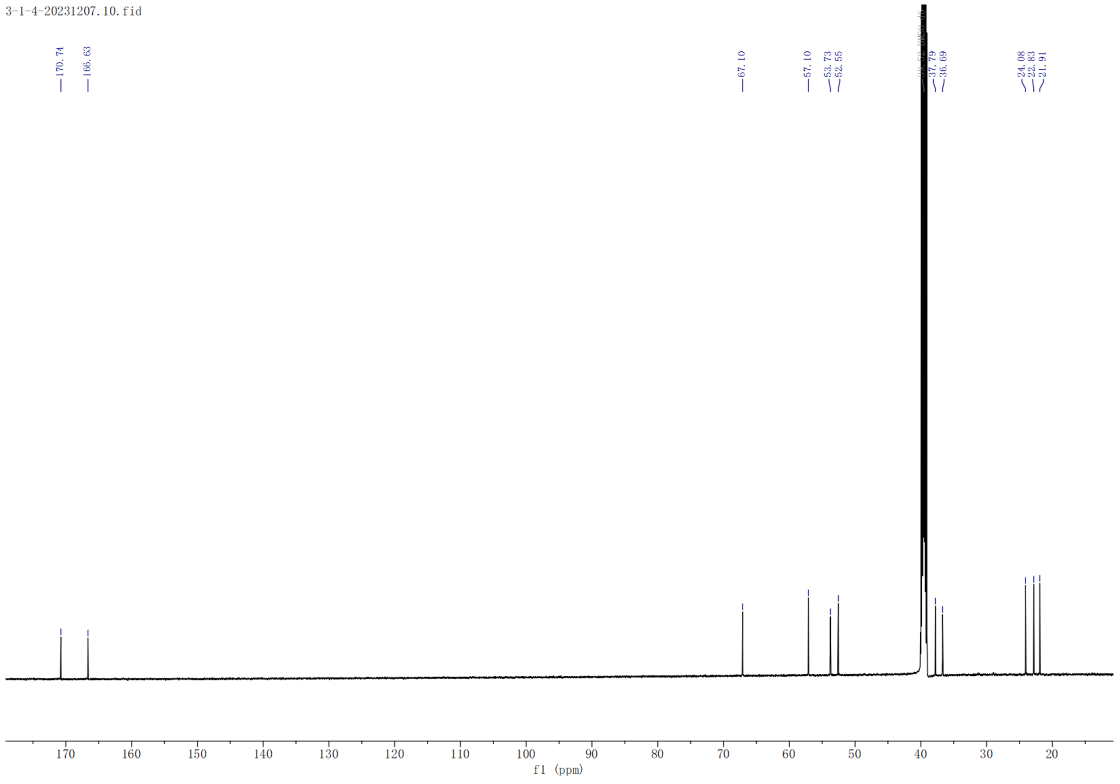

Figure S51. The graphical representation of the separation process.

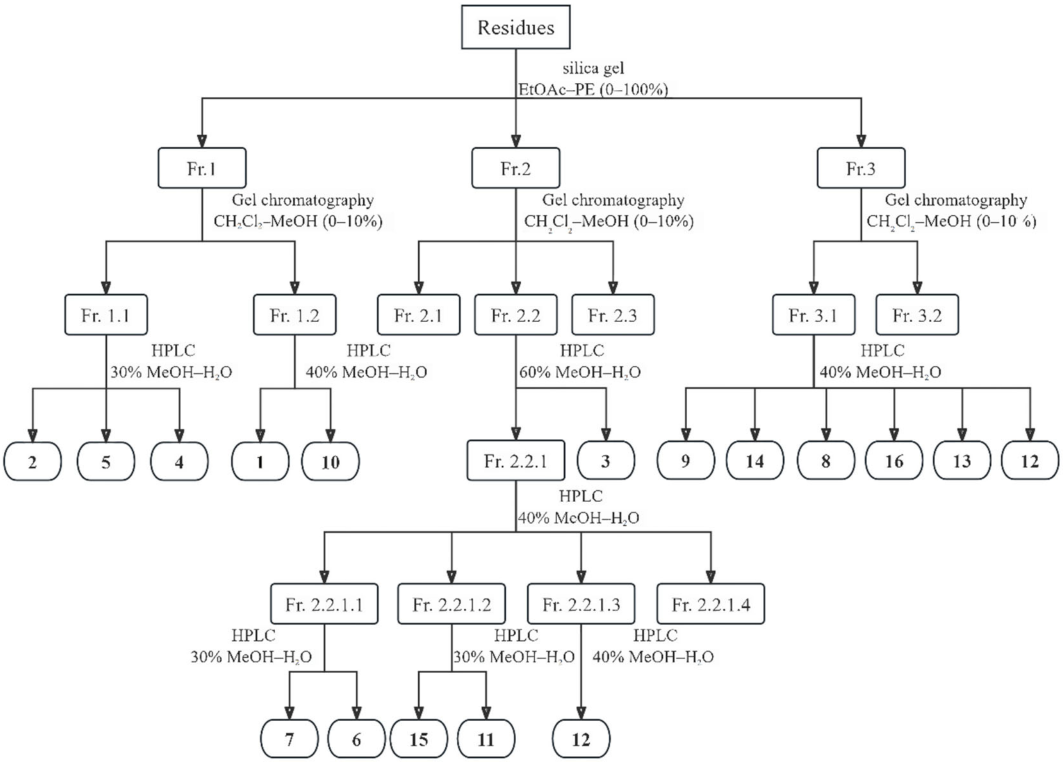

Residues
silica gel
EtOAc–PE (0–100%)
Fr.1
Fr.2
Fr.3
Gel chromatography
CH2Cl2–MeOH (0–10%)
Gel chromatography
CH2Cl2–MeOH (0–10%)
Gel chromatography
CH2Cl2–MeOH (0–10 %)
Fr. 1.1
Fr. 1.2
Fr. 2.1
Fr. 2.2
Fr. 2.3
Fr. 3.1
Fr. 3.2
HPLC
30% MeOH–H2O
HPLC
40% MeOH–H2O
HPLC
60% MeOH–H2O
HPLC
40% MeOH–H2O
2
5
4
1
10
Fr. 2.2.1
3
9
14
8
16
13
12
HPLC
40% MeOH–H2O
Fr. 2.2.1.1
Fr. 2.2.1.2
Fr. 2.2.1.3
Fr. 2.2.1.4
HPLC
30% MeOH–H2O
HPLC
30% MeOH–H2O
HPLC
40% MeOH–H2O
7
6
15
11
12

Table S1. Specific OR of compounds **1–16**.

compounds	Natural	Literature
1	$[\alpha]_D^{20}$-79.8 (c 1.0, MeOH)	+1100 [12]
2	$[\alpha]_D^{20}$+1.95 (c 1.0, MeOH)	$[\alpha]_D^{20}$ +4 (c 0.1, CH_3OH) [14]
3	$[\alpha]_D^{20}$-12 (c 1.0, MeOH)	$[\alpha]_D^{25}$-41.92 (MeOH) [16]
4	$[\alpha]_D^{20}$-34.05 (c 1.0, EtOH)	$[\alpha]_D^{20}$-197 (c0.13, EtOH) [18]
5	$[\alpha]_D^{20}$-9 (c 1.0, MeOH)	$[\alpha]_D^{26}$-85 (c1.0, MeOH) [20]
6	$[\alpha]_D^{20}$-36 (c 1.0, MeOH)	$[\alpha]_D^{20}$ -74 (c 0.05, MeOH) [21]
7	$[\alpha]_D^{20}$-49.95 (c 1.0, MeOH)	-31.3° （c 0.2, MeOH） [23]
8	$[\alpha]_D^{20}$+49.05 (c 1.0, MeOH)	$[\alpha]_D^{20}$ +67 (MeOH) [24]
9	$[\alpha]_D^{20}$-31.95 (c 1.0, EtOH)	$[\alpha]_D^{25}$ -17.0 （c0.1， MeOH） [25]
10	$[\alpha]_D^{20}$-178.05 (c 1.0, MeOH)	$[\alpha]_D^{25}$-78.3 (c0.03, MeOH) [26]
11	$[\alpha]_D^{20}$+58.05 (c 1.0, EtOH)	$[\alpha]_D^{20}$+77.0 (MeOH) [27]
12	$[\alpha]_D^{20}$-28.95 (c 1.0, EtOH)	$[\alpha]_D^{28}$- 24.5° (c0.5, MeOH) [28]
13	$[\alpha]_D^{20}$+43.05 (c 1.0, MeOH)	$[\alpha]_D^{20}$ +67 (MeOH) [29]
14	$[\alpha]_D^{20}$-37.95 (c 1.0, MeOH)	$[\alpha]_D^{20}$-126.1° (c0.26, EtOH) [30]
15	$[\alpha]_D^{20}$+40.95 (c 1.0, MeOH)	$[\alpha]_D^{20}$+128° (c=0.1, EtOH) [18]
16	$[\alpha]_D^{20}$-49.95 (c 1.0, MeOH)	$[\alpha]_D^{20}$-84.77 (c0.53, MeOH) [31]

Table S2. Antibacterial activity of compound **1-16** (50μg/mL).

Compound	Inhibition Rate%				
	Aeromonas hydrophila	*Aeromonas salmonicida*	*Photobacterium angustum*	*Photobacterium halotolerans*	*Vibrio anguillarum*
**1**	-2.1±4.4	17.4±2.5	-1.7±7.7	10.3±3.7	11.7±5.4
**2**	9.7±3.6	-74.8±6.2	-15.9±7.6	-15.9±3.7	14.4±5.7
**3**	-4.6±2.5	-68.3±5.3	-2.6±5.7	0.6±3.7	18.8±1.4
**4**	-12.4±6.8	20.4±1.7	-7.3±0.73	4.8±3.4	20.6±1.0
**5**	-6.7±4.3	26.0±3.0	0.1±6.4	4.8±2.1	15.5±3.6
**6**	13.8±1.0	-65.4±6.5	-4.0±7.2	-15.3±2.0	20.3±1.7
**7**	1.5±1.9	13.1±3.9	0.8±8.0	9.5±2.9	20.9±0.8
**8**	-8.5±4.7	14.2±1.9	0.9±7.7	15.0±4.9	28.7±2.0
**9**	-9.7±2.8	12.9±6.6	-4.0±1.0	17.5±5.7	22.1±1.5
**10**	-14.2±2.6	23.9±2.9	-3.5±0.71	15.3±2.2	16.1±4.0
**11**	-12.4±2.4	29.7±1.3	-4.6±1.4	16.0±0.6	14.6±6.3
**12**	6.2±1.0	-18.5±4.6	1.5±8.6	9.9±2.9	1.9±3.1
**13**	-19.1±3.8	4.1±6.6	-4.0±1.0	15.7±2.2	25.2±2.5
**14**	-10.1±1.7	23.5±3.6	1.0±7.6	15.0±1.5	18.5±1.0
**15**	-16.2±3.6	28.5±0.9	-4.0±1.1	18.3±1.0	22.5±2.3
**16**	-17.7±3.7	17.2±2.2	-3.3±1.6	16.6±2.8	14.1±1.9
Ciprofloxacin	92.4±0.062	89.0±0.030	95.3±0.06	92.8±0.03	93.3±0.03

Table S2. *Cont.*

Compound	Inhibition Rate%				
	Vibrio harveyi	*Pseudomonas aeruginosa*	*Enterobacter cloacae*	*Enterobacter hormaechei*	*Pseudomonas fulva*
**1**	-4.3±4.1	-11.9±9.6	3.5±4.3	-5.4±0.93	26.1±5.1
**2**	-5.8±2.3	-50.0±6.4	-7.5±8.6	-7.7±2.3	21.4±2.3
**3**	-5.7±2.7	-35.5±3.5	6.0±3.3	-7.2±3.0	37.8±6.9
**4**	-3.7±5.7	-22.1±7.5	6.4±1.9	-3.5±5.1	35.2±3.0
**5**	-7.4±3.3	-13.0±1.4	3.7±1.6	-5.2±4.9	14.5±7.5
**6**	0.7±1.7	-10.2±8.1	-3.0±4.0	-1.7±7.9	18.4±5.5
**7**	1.7±0.5	10.3±8.7	-1.8±5.6	-7.3±2.4	35.6±0.5
**8**	2.4±1.8	4.3±3.5	2.3±1.3	-7.1±0.77	44.2±7.7
**9**	1.5±0.6	12.0±5.8	2.9±2.0	-1.6±2.8	1.2±2.3
**10**	2.0±0.7	24.4±3.3	5.6±1.1	-4.6±1.2	20.1±2.2
**11**	0.03±1.4	19.3±3.6	5.1±0.4	-5.0±0.80	21.4±5.2
**12**	3.5±1.8	0.8±3.0	2.1±1.6	-6.5±0.96	28.5±6.0
**13**	1.8±2.3	2.7±10.8	4.6±0.8	-7.7±5.6	35.4±5.9
**14**	2.7±1,5	14.9±0.07	6.6±1.2	-3.9±1.3	11.7±8.8
**15**	1.8±2.0	12.8±3.5	4.4±0.08	-3.0±4.5	14.4±6.1
**16**	-0.02±1.0	12.7±7.4	4.4±0.08	-5.2±2.6	8.1±7.7
Ciprofloxacin	95.4±0.05	94.0±0.02	95.8±0.03	95.4±0.05	95.4±0.030

Table S2. *Cont.*

Compound	Inhibition Rate%				
	Canidia albicans	*Escherichia coli*	*Xanthomonas axonopodi*s	*Staphylococcus aureus*	*Comamonas terrigena*
**1**	9.4±4.8	-35.7±3.6	-23.7±0.56	-8.4±5.3	0.7±3.3
**2**	24.0±7.1	-53.7±4.9	-28.3±2.7	-37.0±4.1	-14.7±7.3
**3**	11.4±4.3	-48.8±6.4	-26.3±0.68	-9.9±3.1	-2.2±0.53
**4**	16.8±3.9	-33.4±4.1	-29.7±0.18	-6.8±5.9	3.2±2.3
**5**	6.6±1.5	-50.4±8.2	-14.0±0.74	-9.2±2.1	4.5±5.1
**6**	19.8±0.8	-47.0±1.5	-38.2±1.5	-18.9±1.9	-16.6±2.6
**7**	5.5±1.1	-30.8±1.8	-20.4±2.7	1.9±0.8	4.0±1.9
**8**	4.8±4.3	-31.9±1.3	-21.3±3.6	1.9±1.6	-4.3±2.9
**9**	2.0±3.9	-32.7±2.9	-29.6±1.6	-3.9±0.47	-4.8±4.5
**10**	8.0±1.3	-33.2±2.0	-23.9±0.69	-5.7±0.98	-0.2±3.4
**11**	2.6±2.7	-29.9±1.0	-19.1±0.82	0.8±1.1	-19.1±2.3
**12**	4.8±3.5	-33.1±0.74	-24.8±1.2	-0.7±1.1	-8.9±6.6
**13**	10.0±2.1	-31.8±2.2	-31.2±0.10	0.7±1.1	1.8±1.5
**14**	5.1±1.8	-30.7±2.8	-36.8±0.28	2.2±0.7	2.4±1.5
**15**	8.4±5.9	-28.8±1.8	-33.2±1.1	0.9±2.4	2.7±1.8
**16**	11.7±2.9	-27.1±2.9	-20.0±0.63	2.1±1.0	4.7±0.9

Ciprofloxacin	93.0±0.05	91.9±0.1	85.4±0.04	90.8±0.07	94.3±0.04

Table S3. Antifungal activity of compound **1-16** (50μg/mL).

Compound	Inhibition Rate%				
	Aspergillus niger	*Fusarium oxysporum*	*Diaporthe citri*	*Alternaria alternata*	*Colletotrichum* sp.
**1**	20.7±8.2	25.6±4.7	23.3±0.052	25.0±9.5	29.6±4.7
**2**	38.1±4.7	36.5±4.7	13.2±8.1	35.6±4.7	19.8±4.8
**3**	24.1±4.7	25.6±9.4	23.3±8.0	28.5±0.020	26.3±0.018
**4**	8.4±2.6	21.9±4.8	26.6±9.4	39.3±8.2	32.9±4.7
**5**	21.9±1.6	29.2±8.2	10.0±4.6	32.2±4.7	29.6±4.7
**6**	16.9±4.5	25.2±4.4	46.5±4.9	17.8±8.2	23.1±9.4
**7**	18.9±6.1	33.0±4.6	36.4±4.7	3.3±47	23.1±4.7
**8**	20.6±8.1	10.3±3.8	26.6±4.7	17.8±8.2	26.3±8.2
**9**	24.2±4.8	22.0±4.7	23.2±0.11	21.4±9.5	26.3±8.2
**10**	36.4±10.9	21.9±9.4	13.2±8.1	25.0±9.4	13.1±4.7
**11**	27.5±4.7	21.9±4.7	23.4±8.2	32.2±4.7	19.8±4.7
**12**	31.0±8.3	14.7±4.7	19.6±4.6	28.3±0.058	26.4±0.025
**13**	23.7±4.0	22.0±4.7	26.5±9.3	28.5±8.1	13.2±9.4
**14**	77.9±7.4	3.0±5.7	23.4±0.12	14.3±4.7	33.0±9.5
**15**	31.0±8.1	25.6±4.7	13.4±0.051	39.2±8.2	72.9±10.0
**16**	6.9±4.7	18.2±8.2	13.4±0.18	17.9±8.2	16.4±0.17
Ciprofloxacin	92.9±0.049	95.0±0.014	93.2±0.10	92.9±0.038	95.5±0.024

Table S4. Acronym list.

Abbreviation	Full Name
NMR	nuclear magnetic resonance
OR	optical rotation
MIC	minimum inhibitory concentration
HRESIMS	high resolution electrospray ionization mass spectroscopy
UV	ultraviolet
1H-1H COSY	1H-1H correlation spectroscopy
HMBC	heteronuclear multiple bond correlation
HPLC	high performance liquid chromatography
EtOAc	ethyl acetate
MeOH	methanol
PE	petroleum ether
